# Grandiosity, vulnerability, and narcissistic fluctuation: Examining reliability, measurement invariance, and construct validity of four brief narcissism measures

**DOI:** 10.3389/fpsyg.2022.993663

**Published:** 2022-10-10

**Authors:** Pentti Henttonen, Juha Salmi, Anssi Peräkylä, Elizabeth A. Krusemark

**Affiliations:** ^1^Department of Psychology and Logopedics, University of Helsinki, Helsinki, Finland; ^2^Department of Social Science, University of Helsinki, Helsinki, Finland; ^3^Department of Neuroscience and Biomedical Engineering, Aalto University, Espoo, Finland; ^4^Department of Psychology and Neuroscience, Millsaps College, Jackson, MS, United States

**Keywords:** grandiose narcissism, vulnerable narcissism, measurement invariance, construct validity, NPI-13, SB-PNI, g-FLUX, HSNS

## Abstract

Continued interest in the distinction between grandiose narcissism, vulnerable narcissism and the fluctuation between grandiose and vulnerable states has expanded the repertoire of self-report instruments. The present study examined the psychometric properties of four brief narcissism measures [the Narcissistic Personality Inventory-13 (NPI-13), Hypersensitive Narcissism Scale (HSNS), Super-Brief Pathological Narcissism Inventory (SB-PNI), and the g-FLUX] in a Finnish sample of university students. Confirmatory factor analyses supported the reliability of the NPI-13, g-FLUX, SB-PNI Vulnerability, and two HSNS subfactors (Oversensitivity and Egocentrism). Tests of measurement invariance indicated the NPI-13, SB-PNI Vulnerability, HSNS Oversensitivity, and the g-FLUX perform similarly between males and females and are generally similar between individuals in younger and older age groups. Construct and predictive validity were evaluated by examining relations between narcissism measures and relevant criteria including psychopathology symptoms, self-esteem, well-being, five factor traits, and empathy. Results supported the construct validity of all four measures, while correlational profiles highlighted the convergence between the g-FLUX and measures of both grandiosity and vulnerability. The NPI-13 was most predictive of NPD symptoms, whereas vulnerable narcissism measures were most predictive of psychopathology. Results further establish the psychometric properties of the NPI-13, SB-PNI Vulnerability, HSNS Oversensitivity, Egocentrism, and provide new validation of the g-FLUX.

## Introduction

Narcissism has stirred a great deal of interest among researchers for many years, partly due to evidence that egocentric traits and reactive, antagonistic behaviors frequently have destructive personal and interpersonal consequences for narcissistic individuals. Narcissistic traits (such as arrogance, superiority, entitlement, need for admiration, and reactivity to criticism) are a group of interrelated traits (Raskin and Hall, 1979; Morf and Rhodewalt, 2001) that parallel symptoms of Narcissistic Personality Disorder (NPD) from the Diagnostic and Statistical Manual of Mental Disorders [DSM: American Psychiatric Association, 1980]. Ongoing research has also emphasized that narcissism is conceptualized as a bidimensional construct, involving both grandiose and vulnerable narcissism (Wink, 1991; Pincus and Lukowitsky, 2010). Grandiose narcissism is associated with a socially-dominant, extraverted, and exploitative personality style, while vulnerable narcissism is characterized by a socially-inhibited, neurotic, and distrusting style (Ronningstam, 2009). Considering the complexity of narcissistic traits, it has become increasingly important to validate the expanding set of narcissism measures that are designed to characterize narcissistic traits and its relations to other personality and psychopathological constructs.

Ongoing theory and research highlight a number of issues regarding the conceptualization and assessment of narcissism. For instance, scholars generally agree that NPD symptoms from the DSM include a combination of traits that are more heavily characterized by grandiosity than vulnerability (Miller et al., 2014). It has been difficult, however, for scholars to reach a consensus on the precise definition of the narcissism construct and there is debate as to what narcissistic traits are adaptive versus pathological. For instance, grandiose narcissism measures assess a combination of adaptive (e.g., leadership, extraversion, and self-esteem: Sedikides et al., 2004; Giacomin and Jordan, 2016) and maladaptive traits (e.g., entitlement, aggression, disagreeableness: Bushman and Baumeister, 1998; Campbell et al., 2002; Miller et al., 2007), whereas vulnerable narcissism and pathological narcissism assessments focus on maladaptive traits including sensitivity to criticism, covert feelings of superiority, neuroticism, and subjective distress (Wink, 1991; Hendin and Cheek, 1997; Pincus et al., 2009). Researchers have also considered whether narcissistic individuals are characteristically grandiose or vulnerable, or whether individuals fluctuate between states of grandiosity and vulnerability from moment to moment (Kernberg, 1998; Ronningstam, 2009; Pincus and Lukowitsky, 2010; Gore and Widiger, 2016). As a result, scholars have developed numerous assessments to capture both grandiose and vulnerable traits and frequently utilize multiple measures to gain a more comprehensive understanding of the range of clinical and behavioral manifestations associated with narcissism.

In keeping with the expanding utility of narcissism research, it is also important to evaluate whether narcissism assessments examine the same construct in different groups such as across cultures, gender, and age groups (e.g., measurement invariance). Examining measurement invariance of narcissism assessments according to gender and age is important considering that narcissism scores differ between men and women (Holtzman et al., 2010; Grijalva et al., 2015) and decrease with age (Foster et al., 2003). To date, only a few studies have examined measurement invariance across age and gender (Wright et al., 2010; Cozma et al., 2014; Morf et al., 2017; Somma et al., 2020) and there are no investigations known to compare measurement invariance across narcissism assessments. Another important validation for these instruments involves examining their construct validity in relation to external correlates relevant to narcissism such as self-esteem, psychopathology, and interpersonal functioning. Given the need to balance experimental goals of utilizing multiple assessments with the practical constraints of limited time and participant attention, researchers have developed several brief self-report assessments of narcissism.

### Brief narcissism measures

A variety of measurements have been developed to assess narcissistic traits, including unitary assessments and multi-dimensional assessments. In the current study, we examine the psychometric properties and construct validity of four brief self-report assessments: (1) the Narcissistic Personality Inventory-13 (NPI-13) as a measure of grandiose narcissism (Gentile et al., 2013), (2) the Hypersensitive Narcissism Scale as a measure of vulnerable narcissism (HSNS: Hendin and Cheek, 1997), (3) the Super-Brief Pathological Narcissism Inventory as a multi-dimensional assessment of pathological narcissism (SB-PNI: Schoenleber et al., 2015), and (4) the g-FLUX as a brief index of narcissistic fluctuation (Oltmanns and Widiger, 2018).

The NPI-13 is an abbreviated, 13-item assessment of grandiose narcissism (Gentile et al., 2013) that contains a total score and subscales based on a three-factor structure (with Leadership/Authority, Grandiose Exhibitionism, and Entitlement/ Exploitativeness). The original NPI was developed to assess narcissistic traits based on DSM-III NPD criteria (Raskin and Hall, 1979) and the NPI-40 is considered the most commonly used assessment of grandiose narcissism. The Hypersensitive Narcissism Scale (HSNS) is a 10-item measure of vulnerable narcissism (Hendin and Cheek, 1997) that assesses sensitivity to criticism, vulnerability, and entitlement. The Super-Brief Pathological Narcissism Inventory (SB-PNI: Schoenleber et al., 2015) contains 12 items that assess both dimensions of pathological grandiose and vulnerable narcissism, reflecting the unidimensional and bidimensional structure of the original PNI (Pincus et al., 2009). Last, the g-FLUX is a 9-item abbreviated version of the FLUX measure of narcissism, which were both designed to assess the degree to which narcissistic individuals fluctuate between self-states of grandiosity and vulnerability (Oltmanns and Widiger, 2018).

### The present study

The primary goal of the current study was to validate the NPI-13, HSNS, SB-PNI, and the g-FLUX. To this end, we examined the psychometric properties and factor structure of each measure in order to determine whether the Finnish translations of each measure were consistent with the properties of their original measures. Consistent with previous research, we expected to replicate the three-factor structure of the NPI-13 (Gentile et al., 2013; Pechorro et al., 2018; Brailovskaia et al., 2019) and we expected a two-factor solution for the Super-Brief PNI (Schoenleber et al., 2015; Morf et al., 2017). We did not have a specific hypothesis regarding the factor structure of the HSNS, as previous studies have reported one, two, and three-factor solutions (Hendin and Cheek, 1997; Fossati et al., 2009). Last, we expected to replicate the unidimensional factor structure of the g-FLUX (Oltmanns and Widiger, 2018). Apart from a small number of Finnish studies examining narcissistic traits (Kalliopuska, 1992; Paunonen et al., 2006; Ojanen et al., 2012), this is also the first study, to our knowledge, to examine narcissistic traits using the NPI-13, HSNS, SB-PNI, and the g-FLUX in a Finnish sample of university students.

We also examined whether each brief narcissism measure could be utilized among both men and women and across age groups. To address this question, we examined the measurement invariance across age and gender for each measure. Considering that NPI-40 scores decrease with age (Foster et al., 2003) and tend to be higher in men (see Grijalva et al., 2015 for a review), we expected similar relationships with NPI-13 scores according to age and gender, but we did not expect violations of measurement invariance. Apart from one study validating a German version of the NPI-13 (Brailovskaia et al., 2019), there are no studies that we know of that have evaluated the measurement invariance of the NPI-13 using the Likert-type response format (Miller et al., 2018b). Previous studies report higher mean PNI Grandiosity in males and higher Vulnerability in females (Wright et al., 2010), with some studies reporting gender invariance at the configural (Wright et al., 2010; Jakšić et al., 2014) and metric level (Morf et al., 2017). Considering the previous evidence for gender invariance along with a recent study demonstrating configural invariance for age using the SB-PNI (Somma et al., 2020); we expected the SB-PNI to achieve configural invariance for gender and age. Considering there is no evidence of age or gender differences on the HSNS of which we are aware (Grijalva et al., 2015), we did not expect violations of measurement invariance. Given the limited research on the g-FLUX, our evaluation of measurement invariance for gender and age was exploratory.

The second goal of our study was to examine the construct and incremental validity of the NPI-13, SB-PNI, HSNS, and the g-FLUX. First, we compared the empirical correlates of the four narcissism measures with two other self-report indices of narcissism and NPD, self-esteem, well-being, symptoms of anxiety and depression, five-factor personality traits, and empathy. Next, we compared the relative contributions of each narcissism measure to each variable using relative weights analyses. Relative weights analyses are useful for comparisons when there is multicollinearity among predictors (Johnson, 2000; Tonidandel and LeBreton, 2011).

As each of the four brief narcissism measures were developed to align with dimensional constructs of grandiose and vulnerable narcissism and aspects of narcissistic fluctuation, we expected the correlations and relative weights to provide a profile of each measure that fit with previous reports. In line with previous evidence for the NPI-13 as a measure of grandiose narcissism, we predicted positive correlations with self-reported NPD symptoms and narcissistic traits, self-esteem, well-being, extraversion; a weaker relationship with measures of vulnerable narcissism; and negative relationships with psychopathology, neuroticism, and aspects of empathy such as Perspective Taking and Empathic Concern. Overall, we expected the SB-PNI to demonstrate positive correlations with measures of grandiose and vulnerable narcissism that reflect its bidimensional structure. We also expected SB-PNI Grandiosity to show positive associations with NPD symptoms psychopathology symptoms and extraversion, but weak to negative correlations with self-esteem, neuroticism, and agreeableness. We expected SB-PNI-Vulnerability to show positive relations with NPD symptoms, anxiety, depression and neuroticism, and negative relationships with self-esteem, agreeableness, and extraversion. Considering contradictory evidence regarding the links between PNI narcissism and empathy, we did not generate predictions for these relationships. We expected the HSNS to correlate positively with other measures of vulnerability but demonstrate weak correlations with grandiosity. We also expected the HSNS to correlate positively with anxiety, depression, neuroticism, and aspects of empathy pertaining to emotional reactivity to others’ distress and exhibit negative associations with self-esteem, well-being, extraversion, and agreeableness, and other aspects of empathy (such as Empathic Concern and Perspective Taking). Last, we expected positive correlations between the g-FLUX and measures of both grandiose and vulnerable narcissism. We also expected the g-FLUX to correlate positively with neuroticism, extraversion, depression, and anxiety and demonstrate negative relationships with self-esteem, well-being, agreeableness, and empathy.

## Materials and methods

### Participants and procedure

Participants were 439 respondents who self-identified as cisgender female (335), cisgender male (90), and individuals identifying as other (14) recruited from university mailing lists [ranging from 18 to 67 years, *M*(*SD*) = 28.8 (8.85)]. Participants were primarily undergraduate students (183), although a portion of participants reported completing bachelor’s degrees (170), master’s degrees (79), and doctoral degrees (7). Individuals were invited to participate if they were over 18 years old and were entered in a raffle for gift cards as compensation. Participants provided informed consent. Prior approval of the study was obtained from the university ethical review board. Each participant completed questionnaires online on a secure research platform used for data collection. Data from two respondents were excluded for having excessive consecutive answers to one or more questionnaires, and one participant had missing data on one or more questionnaires. Descriptive and reliability statistics (ɑ) for the sample are provided in Supplementary Table 1. Two measures were skewed more than 1 (SINS-1: 1.16; GAD-7: 1.03). No measure was kurtotic more than 1.

### Measures

*Narcissistic Personality Inventory-13.* The NPI-13 is a 13-item self-report measure that is a brief adaptation of the original 40-item, forced-choice NPI-40 that measures grandiose narcissism and NPD (Gentile et al., 2013; Miller et al., 2018a). In addition to the total score, the NPI-13 includes three subscales corresponding to Grandiose-Exhibitionism (GE), Leadership-Authority (LA), and Exploitativeness-Entitlement (Ackerman et al., 2011). The Likert type scale includes the more narcissistic item from each of the original forced-choice items (e.g., “If I ruled the world, it would be a better place”/“the thought of ruling the world frightens the hell out of me”). Participants rated their agreement with each item on a 5-point Likert-type scale (1: “strongly disagree,” 5: “strongly agree”). The Finnish version of the NPI-13 was adapted from a previous study using the NPI-40 (Annala, 2015) utilizing items back-translated by three English translation students and modified by an expert board.

#### Hypersensitive narcissism scale

The HSNS is a 10-item self-report measure that examines sensitivity to criticism, vulnerability, and entitlement (Hendin and Cheek, 1997). Each item was rated on a 5-point Likert-type scale (1: “strongly disagree,” 5: “strongly agree”) and participants responded to each item such as “My feelings are easily hurt by ridicule or the slighting remarks of others.” The authors and additional research team members translated and back-translated the items and a bilingual professional checked the items. Similarity between back-translated and original English items were evaluated and adjusted for similarity.

#### Super brief pathological narcissism inventory

The SB-PNI is a 12-item self-report measure (Schoenleber et al., 2015) adapted from the original PNI containing 52 items (Pincus et al., 2009). The SB-PNI contains two 6-item subscales corresponding to the second-order factors of grandiosity (SB-PNI-G) and vulnerability (SB-PNI-V). Each item was rated on a 6-point Likert-type scale (0: “not at all like me,” 5: “very much like me”) and participants responded to items such as “It’s hard to feel good about myself unless I know other people admire me.” Finnish items were adapted from the original PNI independently translated for an unpublished study by one of the authors (AP) and another researcher, modified according to backtranslation by an expert and tested for comprehensibility on 23 native Finnish speakers.

*g-FLUX.* The g-FLUX is a 9-item self-report measure of narcissistic fluctuation adapted from the original FLUX measure (Oltmanns and Widiger, 2018). The g-FLUX contains three subscales corresponding to fluctuations between Indifference-Anger (I), Grandiosity-Shame (G), and Assertiveness-Insecurity (A). Each item describes the tendency to switch between states of grandiosity and vulnerability (“e.g., My thoughts shift between expectations of greatness and expectations of total failure”). Participants rated items on a 5-point Likert-type scale (1: “strongly disagree,” 5: “strongly agree”). The Finnish version of the g-FLUX was translated by PH and two other researchers.

#### Personality disorder questionnaire-4

The PDQ-4 is a 99-item self-report assessment of DSM-IV/V personality disorder traits (Hyler, 1994). The section corresponding to NPD containing 9 items were included (PDQ-NPD). Each item contains a true (1) or false (0) response option and participants responded to items such as “I often find myself thinking about how great a person I am, or will be.”

*Single Item Narcissism Scale.* The SINS (Konrath et al., 2014) is a self-report assessment of narcissism, containing one response item rated on a 7-point Likert-type scale (1: “not very true of me,” 7: “very true of me”). Participants rated the degree to which they agreed with the statement “I am a narcissist,” accompanied by a brief definition of narcissist as an egotistical, self-focused, and vain individual.

*Interpersonal Reactivity Index.* The IRI is a 28-item self-report scale examining empathy (Davis, 1983). It contains four 7-item subscales: Perspective-Taking (PT), Empathic Concern (EC), Fantasy (F), and Personal Distress (PD). Each item was rated on a 5-point Likert scale (0: “does not describe me,” 4: “describes me very well”) and participants rated their agreement with items such as “I often have tender, concerned feelings for people less fortunate than me.”

#### Empathy quotient-short version

The EQ-Short is a 22-item self-report measure of empathizing (Wakabayashi et al., 2006). Items were rated on a 4-point scale ranging from “strongly agree” to “strongly disagree,” in which item scores range from 0 to 2 in magnitude. Participants rated their agreement with items such as “I can pick up quickly if someone says one thing but means another.”

#### Patient health questionnaire-9

The PHQ-9 is a self-report measure of depressive symptoms, corresponding to the DSM-IV criteria (Kroenke et al., 2001). Participants rated the degree and frequency for which they experienced symptoms over the course of 2 weeks from 0 (not at all) to 3 (nearly every day). Participants rated the frequency for which they experienced problems with issues such as feeling down or hopeless, feeling tired, or experiencing anhedonia.

#### Generalized anxiety disorder scale

The GAD-7 is a 7-item self-report assessment of generalized anxiety symptoms (Spitzer et al., 2006). Participants rated the degree and frequency for which they experienced symptoms over the course of 2 weeks from 0 (not at all) to 3 (nearly every day). Participants rated the frequency for which they experienced problems with issues such as feeling unable to stop or control worrying or having trouble relaxing.

#### Rosenberg self-esteem scale

The RSE is a 10-item self-report measure of self-esteem (Rosenberg, 1965). Each item was rated on a 5-point Likert scale (1: “strongly disagree,” 5: “strongly agree”). Participants rated the degree to which they agreed with statements such as “I feel like a person who has a number of good qualities.”

#### World Health Organization—Well-being index

WHO-5 is a 5-item self-report measure of current mental well-being. Participants rate the degree to which they experienced positive well-being over the course of 2 weeks on a 6-point scale (0: “at no time,” 5: “all of the time”: Topp et al., 2015), including statements such as “I have felt active and vigorous.”

#### Short five

S5 contains 60 items that directly examine the five-factor model of personality (Konstabel et al., 2012). Each subscale contains 12 items corresponding to standard NEO PI-R structure of Openness (O), Conscientiousness (C), Extraversion (E), Agreeableness (A), Neuroticism (N). For this study, we only used E, A, and N as they are most relevant to narcissistic traits. Each item was rated on a 7-point Likert scale (−3: “strongly disagree,” 3: “strongly agree”), and participants rated their agreement with statements such as “I am often nervous, fearful, and anxious, and I worry that something might go wrong.”

### Data analytic strategy

Analyses were conducted with R 4.1.0 and Lavaan 0.6–9 package (Rosseel, 2012). First, confirmatory factor analysis was performed by building and comparing alternative models to assess data fit. For all four narcissism scales, a single factor model was compared to a correlated factor model and an orthogonal confirmatory bifactor model. In contrast to the correlated factor model, using the bifactor model allows for separation of the variance contributions due to a general factor and subfactors. Supplementing fit indices, a good model is defined by an adequate degree of unidimensionality, despite the multidimensional contributions of the subfactors (Rodriguez et al., 2016). All scales exhibited deviations from multiple normality, assessed with Mardia’s (1970) test. Scale values were thus treated as ordinal and DWLS estimator was used, as recommended in cases with less than seven categories in Likert scale (Li, 2016). Robust standard errors and mean- and variance-adjusted test statistics are reported. Model fit was assessed by comparing scaled Satorra-Bentler χ^2^, CFI, TLI, RMSEA, and SRMR goodness-of-fit statistics. For CFI and TLI indices, values greater than 0.90 were interpreted to exhibit a good fit and values greater than 0.95 excellent fit. RMSEA and SRMR values smaller than 0.08 indicate a reasonable fit and values smaller than 0.05 a good fit (Hu and Bentler, 1999). Factor loadings were calculated for all models and scales. Factor loadings above 0.50 were interpreted as satisfactory and above 0.70 as ideal. Dimensionality of the confirmatory bifactor model was determined by comparing omega hierarchical (ωH) of the common factor and subscales, along with explained common variance (*ECV*) due to factor or item and percentage of uncontaminated correlations (*PUC*). Invariance tests were conducted and reported for the correlational model of each narcissism scale between males and females and median-split age groups (≤25 vs. >25) according to Putnick and Bornstein (2016). Correlational models were used for invariance tests due to issues with fitting and interpretation of bifactorial model invariance. For purposes of comparing loadings and intercepts in separate steps, data was treated as continuous and maximum likelihood estimation with robust standard errors (MLR) was used, which has been shown to perform roughly equal to WLSMV estimation in variables with more than three categories (Beauducel and Herzberg, 2006). For each measure, the configural model was compared to metric (factor scores constrained) and scalar (intercepts constrained) models. When significant differences in fit were detected, models were altered by releasing loading or intercept constraints in a backwards fashion according to modification indices. If full or partial scalar invariance with limited differences in loadings was established, latent means were interpreted to be reliable and subsequently compared between groups (Steinmetz, 2013).

As part of our validation of each brief narcissism measure, we then examined the intercorrelations and average inter-item correlations from the four narcissism scales. We then evaluated construct validity using bivariate correlations with self-reported NPD symptoms, trait narcissism, psychopathology symptoms, self-esteem, well-being, five factor personality traits, and empathy^1^. In addition, we examined the similarity of their empirical profiles using double-entry *Q* correlations (McCrae, 2008). Last, we examined incremental validity using relative weights analyses (Johnson, 2000; Tonidandel and LeBreton, 2011) to demonstrate the relative proportion of variance that each narcissism measure contributed to each criterion variable. Relative weights analyses quantify the relative contribution each measure makes to *R^2^* and provide important information about the relative predictive validity among interrelated variables. These analyses supplement hierarchical and incremental regressions that credit explanatory variance according to the order of added variables, leading to possible misinterpretation on variables’ true contributions.

## Results

### Model comparison

As the first goal of our study involved evaluating the psychometric properties of the four brief narcissism measures (the NPI-13, SB-PNI, HSNS, and the g-FLUX), we conducted confirmatory factor analysis, using comparative model fitting for each measure. Detailed fit indices for the compared models are listed in Table 1. As expected, the correlated model exhibited a better fit over the single factor model for all four narcissism scales in terms χ^2^ and reported fit indices. However, none of the correlational models were indicative of a good fit, with the exception of g-FLUX, for which the correlational model exhibited excellent (CFI, TLI > 0.95; SRMR < 0.05) or good (RMSEA < 0.08) fit. The NPI-13 and HSNS both demonstrated excellent fit with the bifactor models (CFI > 0.95; SRMR < 0.05; RMSEA < 0.08). The SB-PNI showed the closest fit to the bifactor model, although the fit indices supported a good fit for CFI (0.932) and SRMR (0.061) and below threshold for good fit in terms of TLI (0.894) and RMSEA (0.110)^2^. The bifactor model for the g-FLUX did not converge. To obtain fit and dimensionality indices, S-1 bifactor model was estimated instead, dropping the Assertiveness/Insecurity subfactor from the model (Eid et al., 2017). This model for g-FLUX demonstrated excellent fit (CFI, TLI > 0.95; SRMR < 0.05; RMSEA < 0.08), though inferior to correlational model in all indices.

**Table 1:** CFA fit indices of single factor, correlated factors and bifactor models.

Scale	Model	χ^2^	df	CFI	TLI	RMSEA	SRMR
NPI-13	1-factor	1090.65	65	0.743	0.692	0.190	0.147
	Correlated	589.14	62	0.868	0.834	0.139	0.104
	Bifactor	111.70	52	0.985	0.978	0.051	0.041
SB-PNI	1-factor	952.12	54	0.728	0.667	0.195	0.120
	Correlated	665.35	53	0.814	0.769	0.162	0.108
	Bifactor	265.56	42	0.932	0.894	0.110	0.061
HSNS	1-factor	464.54	27	0.557	0.409	0.192	0.142
	Correlated	229.03	26	0.794	0.715	0.134	0.106
	Bifactor	48.24	18	0.969	0.939	0.062	0.046
g-FLUX	1-factor	283.03	27	0.901	0.868	0.147	0.081
	Correlated	63.06	24	0.985	0.977	0.061	0.038
	Bifactor^*^	66.87	21	0.982	0.970	0.071	0.039

NPI-13, Narcissistic Personality Inventory-13; SB-PNI, Super-Brief Pathological Narcissism Inventory; HSNS, Hypersensitive Narcissism Inventory; CFI, comparative fit index; TLI, Tucker-Lewis index; RMSEA, root mean square of approximation; SRMR, standardized root mean square residual.

* g-FLUX Bifactor model estimated with S-1 model (omitting A factor).

Factor loadings and bifactor indices are listed in Table 2. All NPI-13 items had bifactor loadings (λ ≥ 0.30) on the general factor, with the exception of item 8 (λ = 0.27). Bifactor item loadings for the three subfactors were greater than 0.20, with the exception of two items from on the GE subfactor (2,8), two items on the LA subfactor (3,6), and one item from the EE subfactor (1). The reliability was high (ω = 0.91) for the general factor as well as for LA and GE subfactors (ω > 0.80). The EE subfactor’s consistency was acceptable (ω > 0.70). Negative loadings in bifactorial subfactors were observed in LA (items 3 and 6) and EE (item 1). Omega hierarchical for the NPI-13 general factor was 0.78 and *ECV* was 0.59. According to Reise et al. (2013), ωH above 0.70, *ECV* above 0.60, and *PUC* values < 0.80 can be interpreted as thresholds for unidimensionality. The NPI-13 general factor’s indices are very close to this limit, and in combination with the attained *PUC* value (0.72), the factor structure of NPI-13 can be interpreted as essentially unidimensional. This is also supported by the relatively high number of unidimensional items (6), indicated by high I-*ECV* (>0.80). Parameters for the EE and LA subfactors did not exhibit large degrees of independence from the general factor (ωH < 0.10, *ECV* < 0.10). Items 2 and 11 of the GE subfactor exhibited low contribution to the general factor (λ < 0.20 and I**-***ECV* < 0.30). These items caused the GE subfactor to exhibit relative independence from the general factor (ωH = 0.55, *ECV* = 0.28). Covariance between the NPI-13 subfactors also supported the distinction between the GE factor from the other two factors, as well as a strong correlation between LA and EE.

**Table 2:** Factor loadings and bifactor indices of narcissism scales and subscales.

A. NPI-13 (PUC = 0.72)						B. SB-PNI (PUC = 0.55)				
Item	λg	λGE	λLA	λEE	ECV	Item	λg	λG	λV	ECV
1	0.51 (0.60)			0.58 (−0.19)	0.91	22	0.66 (0.72)	0.72 (−0.40)		0.76
4	0.58 (0.62)			0.67 (0.44)	0.67	26	0.46 (0.44)	0.48 (0.25)		0.76
7	0.43 (0.46)			0.50 (0.23)	0.80	31	0.57 (0.57)	0.64 (0.43)		0.63
10	0.52 (0.56)			0.61 (36)	0.70	33	0.66 (0.75)	0.73 (−0.41)		0.77
3	0.70 (0.60)		0.79 (−0.21)		0.94	42	0.49 (0.48)	0.55 (0.41)		0.58
6	0.72 (0.79)		0.80 (−0.10)		0.99	45	0.64 (0.64)	0.72 (0.58)		0.55
9	0.59 (0.61)		0.67 (0.51)		0.59	8	0.60 (0.51)		0.64 (0.35)	0.69
12	0.63 (0.66)		0.72 (0.48)		0.65	17	0.51 (0.24)		0.56 (0.66)	0.12
2	0.42 (0.42)	0.45 (0.14)			0.90	30	0.75 (0.68)		0.79 (0.33)	0.81
5	0.73 (0.36)	0.80 (0.72)			0.20	32	0.71 (0.52)		0.76 (0.57)	0.46
8	0.79 (0.27)	0.87 (0.92)			0.08	36	0.73 (0.56)		0.78 (0.54)	0.52
11	0.59 (0.60)	0.64 (0.19)			0.91	50	0.49 (0.25)		0.53 (0.59)	0.16
13	0.73 (0.33)	0.82 (0.76)			0.16					
ω	0.87 (0.91)	0.82 (0.84)	0.80 (0.86)	0.65 (0.71)		ω	0.86 (0.90)	0.78 (0.84)	0.82 (0.85)	
ωH	0.78	0.55	0.05	0.09		ωH	0.72	0.05	0.46	
ECV	0.59	0.28	0.08	0.06		ECV	0.58	0.17	0.25	
GE^±^			0.49	0.52		G^±^			0.66	
LA^±^				0.90						
**C. HSNS. (PUC = 0.56)**					**D. g-FLUX(PUC = 0.83)**					
**Item**	**λg**	**λOS**	**λEGO**	**ECV**	**Item**	**λg**	**λI**	**λG**	**λA** ^*^	**ECV**
1	0.45 (0.24)	0.47 (0.42)		0.25	1 (I3)	0.65 (0.54)	0.79 (0.63)			0.42
2	0.60 (−0.03)	0.66 (0.81)		0.00	4 (I8)	0.62 (0.52)	0.76 (0.54)			0.49
3	0.52 (0.29)	0.52 (0.29)		0.29	7 (I9)	0.47 (0.39)	0.56 (0.37)			0.53
6	0.32 (0.64)	0.23 (0.64)		0.99	2 (G3)	0.71 (0.64)		0.75 (0.40)		0.72
7	0.83 (0.31)	0.91 (0.79)		0.13	5 (G5)	0.74 (0.68)		0.78 (0.34)		0.80
4	0.35 (0.45)		0.46 (0.16)	0.89	8 (G8)	0.79 (0.72)		0.83 (0.45)		0.72
5	0.35 (0.39)		0.72 (0.68)	0.25	3 (A3)	0.58 (0.62)			0.61	
8	0.32 (0.30)		0.46 (0.11)	0.96	6 (A4)	0.67 (0.72)			0.71	
10	0.30 (0.31)		0.62 (0.61)	0.20	9 (A7)	0.68 (0.73)			0.71	
ω	0.66 (0.78)	0.64 (0.75)	0.61 (0.69)		ω	0.85 (0.89)	0.73 (0.75)	0.80 (0.83)	0.67	
ωH	0.42	0.55	0.31		ωH	0.80	0.40	0.21		
ECV	0.36	0.43	0.22		ECV	0.73	0.17	0.10		
OS^±^			0.26		I^±^			0.57	0.72	
					G^±^				0.89	

Factor loadings and g factor loadings presented, bifactor loadings in parentheses. ECV, Explained common variance; PUC, Percentage of uncontaminated correlations; ωH, Omega hierarchical for scale or subscale; GE, Grandiose/Exhibitionism; LA, Leadership/Authority; EE, Entitlement/Exploitativeness; G, Grandiosity; V, Vulnerability; I, Indifference/Anger; G, Grandiosity/Shame; A, Assertiveness/Insecurity; OS, Oversensitivity to Judgment; EGO, Egocentrism.

* g-FLUX bifactor model estimated with S-1 model without A subfactor. ±Factor covariances.

Bifactor loadings for the SB-PNI items were >0.30 on the general factor, with the exception of items 17 and 50. Half of the bifactor loadings for SB-PNI Grandiosity exceeded 0.40, although negative loadings on the bifactorial Grandiosity subfactor were observed for items 22 and 33. All bifactor loadings for the Vulnerability subfactor were >0.30. Reliability of the general factor was high (ω = 0.90), and good for SB-PNI Grandiosity (ω = 0.84), and SB-PNI Vulnerability (ω = 0.85). Covariance between the Grandiosity and Vulnerability subfactors was moderate (0.66). Low ωH for the Grandiosity subfactor suggested it was mainly explained by the general factor. Items 17 and 50 of the Vulnerability subfactor had very low contribution to the general factor (λ ≤ 0.30 and I**-***ECV* < 0.20). The Vulnerability subfactor exhibited relative independence from the general factor (ωH = 0.46, *ECV* = 0.25). Considering the general factor accounts for most of the variance (ωH = 0.72, *ECV* = 0.58) along with the percentage of uncontaminated correlations (0.55), the factor structure of SB-PNI can be interpreted as somewhat unidimensional.

Bifactor loadings for the HSNS items were ≥0.30 on the general factor, with the exception of items 1, 2, and 3. Bifactor loadings for the OS subfactor were >0.40 (exempt from item 3), whereas loadings for the EGO subfactor were >0.60 (exempt from items 4 and 8). The reliabilities of the general factor (ω = 0.78) and the Oversensitivity (OS) subfactor were acceptable (ω = 0.75), and the Egocentrism (EGO) subfactor just below an acceptable threshold: (ω = 0.69). Covariance between the OS and EGO subfactors was modest (0.26). Both the OS and EGO subfactors exhibited moderate independence from the general factor (ωH > 0.30), with OS having the larger indices (ωH = 0.55, *ECV* = 0.43) than the general factor (ωH = 0.42, *ECV* = 0.36). Considering the parameters of the HSNS general factor mentioned above along with the percentage of uncontaminated correlations (0.56), the HSNS cannot be interpreted as unidimensional.

The g-FLUX items had substantial bifactor loadings (λ ≥ 0.50) on the general factor with the exception of item 7 (I-9). Reliability of the general factor was high (ω = 0.89), the I and G subfactors were acceptable (ω > 0.75), and the A subfactor was adequate (ω = 0.67). Covariance between the three subfactors was moderate to high. Given that the g-FLUX bifactorial model did not converge, another bifactor model was fit using two of the three subfactors (omitting the Anger-Indifference (A) subfactor). Bifactor loadings from the I and G subfactors were above 0.30. Parameters for the general factor supported unidimensionality of the g-FLUX (ωH = 0.80, *ECV* = 0.73, *PUC* = 0.83).

Overall, the confirmatory factor analysis and comparative model fitting demonstrated that both the NPI-13 exhibited the closest fit with the bifactor model and evidence of reliability as unidimensional scale as well as with its three subfactors. The SB-PNI exhibited the closest fit with the bifactor model and demonstrated good levels of reliability for both the general and subfactors. Separate single factor model fit for the respective SB-PNI subfactors, however, revealed poor model fit for SB-PNI Grandiosity and excellent model fit for SB-PNI Vulnerability. The HSNS demonstrated the closest fit with a bifactor model, showing adequate reliability for the OS and EGO subfactors. The g-FLUX demonstrated the closest fit with the correlated factor model, demonstrating adequate to good reliability for the three subfactors. Re-estimation with the S-1 bifactor model for the g-FLUX demonstrated excellent fit using the two subfactors (Indifference/Anger and Grandiosity/Vulnerability).

### Measurement invariance

Additional evaluation of the four brief narcissism measures examined whether each measure exhibited measurement invariance across gender and age groups. Measurement invariance tests were run on the correlated factor models (while treating data as continuous). Results are detailed in Table 3. Loadings, intercepts, and means were compared between self-identifying cisgender males (n = 90) and cisgender females (n = 335) and between groups of individuals ages 25 years or younger (*n* = 206) and older (*n* = 233) than the median age of 25.

**Table 3:** Invariance test fit indices between configural, metric and scalar models for age and gender.

Variable		Model	df	χ^2^	Comp. to	p(χ^2^)	CFI (Δ CFI)	TLI (Δ)	RMSEA(Δ)	SRMR(Δ)
Gender	NPI-13	Configural	124	411.945			0.828	0.784	0.109	0.103
		Metric	134	418.484	Configural	0.676	(0.002)	(−0.022)	(0.018)	(0.002)
		Scalar	144	439.198	Metric	**0.033** ^*^	(−0.005)	(0.008)	(−0.002)	(0.001)
		Scalar^1^	143	429.529	Metric	0.371	(0.000)	(0.012)	(−0.003)	(0.001)
	SB-PNI	Configural	106	527.596			0.748	0.686	0.138	0.100
		Metric	116	519.302	Configural	0.486	(0.000)	(0.028)	(−0.006)	(0.006)
		Scalar	126	547.009	Metric	**0.004** ^**^	(−0.009)	(0.013)	(−0.003)	(0.002)
		Scalar^2^	124	530.370	Metric	0.327	(−0.001)	(0.018)	(−0.004)	(0.000)
	g-FLUX	Configural	48	79.741			0.970	0.955	0.058	0.051
		Metric	54	81.608	Configural	0.980	(0.004)	(0.011)	(−0.007)	(0.001)
		Scalar	60	100.063	Metric	**0.005** ^**^	(−0.011)	(−0.010)	(0.007)	(0.005)
		Scalar^3^	58	87.367	Metric	0.220	(−,002)	(0.001)	(0.000)	(0.002)
Age	NPI-13	Configural	124	418.187			0.832	0.788	0.109	0.103
		Metric	134	432.965	Configural	0.108	(−0.003)	(0.012)	(−0.003)	(0.006)
		Scalar	144	454.818	Metric	**0.022** ^*^	(−0.006)	(0.008)	(−0.002)	(0.001)
		Scalar^4^	143	434.494	Metric	0.999	(−0.003)	(0.009)	(−0.003)	(0.002)
	SB-PNI	Configural	106	507.278			0.754	0.694	0.134	0.099
		Metric	116	497.328	Configural	0.767	(0.003)	(0.030)	(−0.007)	(0.005)
		Scalar	126	536.888	Metric	**0.001** ^***^	(−0.016)	(0.005)	(−0.001)	(0.000)
		Scalar^5^	124	515.331	Metric	0.087	(−0.010)	(0.007)	(−0.001)	(0.001)
	HSNS	Configural±	52	184.416			0.784	0.701	0.108	0.092
		Metric	59	191.624	Configural	0.234	(−0.004)	(0.003)	(−0.006)	(0.004)
		Scalar	66	203.657	Metric	0.097	(−0.009)	(0.019)	(−0.004)	(0.002)
	g-FLUX	Configural	48	79.620			0.971	0.956	0.058	0.038
		Metric	54	88.161	Configural	0.214	(−0.002)	(0.002)	(−0.002)	(0.010)
		Scalar	60	95.405	Metric	0.320	(−0.001)	(0.003)	(−0.002)	(0.002)

Models with constraints on intercepts lifted identified with superscript. Bold values indicate significant fit differences between compared models. *p < 0.05; **p < 0.01; and ***p < 0.001.

1 NPI-13: item 4.

2 SB-PNI: Items 32 and 42.

3 g-Flux: Item 6(A4) and 8(G8).

4 NPI-13: item 13.

5 SB-PNI: Items 8 and 17. ±Configural model with Gender in HSNS did not converge.

The NPI-13 demonstrated gender invariance at the metric level. After releasing the equality constraints on item 4, belonging to EE factor, partial scalar invariance was attained. Males had higher latent scores on EE (estimate = 0.38, *z* = 3.41, *p* < 0.001) and trend of higher latent scores on LA (estimate = 0.21, *z* = 1.83, *p* = 0.07). SB-PNI demonstrated gender invariance at the metric level. Partial scalar invariance was achieved by releasing constraints on the intercepts of item 32 (Vulnerability) and item 42 (Grandiosity). There were no differences in latent subfactor scores. The configural model for HSNS total with regards to gender did not converge. Respective tests of HSNS OS and EGO single factor models demonstrated that only the OS subfactor could be successfully estimated to achieve metric invariance according to gender (see Supplementary material). The g-FLUX demonstrated gender invariance at the metric level and partial scalar invariance after releasing intercept constrains on items 6 (A) and 8 (G), with no differences in latent subfactor scores.

The NPI-13 achieved metric variance according to age. Once constraints on one NPI-13 item loading (item 13, GE) were lifted, partial scalar invariance was also established. The younger age group demonstrated higher latent scores on the GE subfactor (estimate = 0.09, *z* = 2.42, *p* < 0.05). The SB-PNI achieved metric invariance as a function of age. Partial scalar invariance was achieved once constraints on intercepts of two items (8 and 17) belonging to the Vulnerability subfactor were lifted. Intercepts were lower (2.2 vs. 2.6) for item 1 and higher (2.9 vs. 2.6) for item 2 in the older age group. The younger age group exhibited trending higher latent scores on SB-PNI Grandiosity (estimate = 0.18, *z* = 1.88, *p* = 0.06). Scalar invariance was established for HSNS total in terms of age. Respective tests of the HSNS OS and EGO factors demonstrated that the OS subfactor achieved metric invariance whereas the EGO subfactor achieved scalar invariance according to gender (see Supplementary material). There were no significant differences in latent HSNS factor scores as a function of age. Scalar invariance was established for the g-FLUX as a function of age, with no differences in latent subfactor scores.

In summary, measurement invariance analyses according to gender revealed that the NPI-13, SB-PNI, HSNS OS and the g-FLUX achieved metric invariance. Releasing constraints on items from the NPI-13, SB-PNI, and the g-FLUX enabled partial scalar invariance across gender groups. With regard to age, measurement invariance analyses revealed that the NPI-13 and SB-PNI achieved metric invariance and partial scalar invariance once item constraints were lifted. The HSNS, HSNS EGO, and the g-FLUX achieved scalar invariance as a function of age.

### Relations among brief narcissism scales

The second goal of our study involved examining the construct validity of each measure. To this end, we first examined correlations among the four narcissism measures. The four brief narcissism measures^3^ were all generally interrelated (with an average intercorrelation of *r* = 0.30)^4^ and exhibited a broad range of correlations from 0.03 (NPI-13 − HSNS OS) to 0.60 (SB-PNI Vulnerability – HSNS OS: see Table 4). The NPI-13 exhibited modest correlations with the brief indices of narcissistic vulnerability, including SB-PNI Vulnerability (*r* = 0.14), and HSNS EGO (*r* = 0.22). Alternatively, the measures of narcissistic vulnerability were moderately interrelated, with SB-PNI Vulnerability exhibiting a moderate correlation with HSNS OS (*r* = 0.60). The two HSNS factors (OS and EGO) were only modestly interrelated (*r* = 0.25). Last, the g-FLUX demonstrated somewhat moderate correlations with all of the brief narcissism measures, showing the lowest relation with HSNS OS (*r* = 0.39), followed by the NPI-13 (r = 0.43), and SB-PNI Vulnerability (*r* = 0.50).

**Table 4:** Intercorrelations between brief narcissism scales, subscales, and age.

	Variable	**1**	2	3	4	**5**	6	7	**8**	9	10	**11**	12	13	14
**NPI-13 total**	**1**	(0.29)													
Leadership/authority (LA)	2	***0.83***	(0.45)												
Grandiose/exhibitionism (GE)	3	***0.79***	*0.42*	(0.37)											
Entitlement/exploitativeness (EE)	4	***0.82***	***0.65***	*0.40*	(0.30)										
**SB-PNI total**	**5**	**0.28**	0.18	0.21	0.29	(0.30)									
SB-PNI grandiosity	6	*0.35*	*0.32*	0.23	*0.33*	***0.85***	(0.30)								
PNI vulnerability	7	0.14	0.01	0.15	0.19	***0.89***	***0.52***	(0.42)							
**HSNS total**	**8**	**0.16**	0.02	0.12	0.25	***0.51***	*0.31*	***0.55***	(0.17)						
Oversensitivity to judgment (OS)	9	0.03	−0.09	0.06	0.10	***0.55***	*0.34*	***0.60***	***0.83***	(0.25)					
Egocentrism (EGO)	10	0.22	0.14	0.12	0.29	0.16	0.10	0.17	***0.72***	*0.25*	(0.26)				
**g-FLUX total**	**11**	***0.43***	*0.32*	0.25	*0.49*	***0.56***	*0.47*	***0.50***	*0.49*	*0.39*	0.35	(0.36)			
Indifference/anger (I)	12	*0.32*	0.27	0.15	*0.39*	0.27	0.22	0.25	*0.34*	0.23	0.28	***0.76***	(0.43)		
Grandiosity/shame (G)	13	*0.32*	0.21	0.20	*0.37*	***0.62***	***0.54***	***0.54***	*0.46*	*0.39*	0.29	***0.86***	*0.42*	(0.57)	
Assertiveness/insecurity (A)	14	*0.43*	*0.32*	0.28	*0.46*	*0.46*	*0.37*	*0.42*	*0.41*	*0.33*	0.28	***0.85***	*0.50*	***0.64***	(0.39)
Age	**15**	−0.14	−0.08	−0.16	−0.09	−0.28	−0.29	−0.21	−0.11	−0.15	0.00	−0.08	−0.05	−0.18	−0.09

*N* = 439. Average inter-item correlation on diagonal in parentheses. Coefficients above 0.10 are significant at *p* < 0.05. Coefficients above 0.16 are significant at *p* < 0.001. Coefficients 0.30 or above italicized, coefficients ≥ 0.50 in bold.

### Construct validity

Bivariate correlations were utilized to examine the construct validity of the four brief narcissism measures and their associations with two other self-report narcissism instruments, psychopathology symptoms, self-esteem, well-being, five factor personality traits, and empathy (Table 5). First, the NPI-13 demonstrated positive correlations with self-reported NPD and narcissism (PDQ-NPD: *r* = 0.41; SINS: *r* = 0.43), extraversion (*r* = 0.29), and self-esteem (*r* = 0.26) and negative correlations with agreeableness and Empathic Concern (*r’s* = −0.45 and − 0.26, respectively). By contrast, SB-PNI Vulnerability exhibited (but somewhat weaker) positive correlations with self-reported NPD and narcissism (PDQ-NPD: *r* = 0.30; SINS: *r* = 0.21), positive correlations with depression, anxiety, and Personal Distress (*r*’s from 0.40 to 0.45), a moderate relationship with neuroticism (*r* = 0.61), and negative correlations with self-esteem (*r* = −0.53), subjective well-being (*r* = −0.33), and extraversion (*r* = −0.20). HSNS OS also exhibited positive correlations with PDQ-NPD symptoms (*r* = 0.27), depression (*r* = 0.42), anxiety (*r* = 0.45), Fantasy (*r* = 0.27), and Personal Distress (*r* = 0.41), a moderate association with neuroticism (*r* = 0.63), and negative associations with self-esteem, well-being, and extraversion (*r*’s from −0.28 to −0.47). HSNS EGO exhibited positive correlations with self-reported NPD and narcissism (PDQ-NPD: *r* = 0.28; SINS: *r* = 0.35) and a modest association with neuroticism (*r* = 0.17), along with negative correlations with extraversion (*r* = −0.31), agreeableness (*r* = −0.52), Empathic Concern (*r* = −0.44), Perspective Taking (*r* = −0.26), and EQ Empathy (*r* = −0.35). Finally, the g-FLUX demonstrated positive correlations with self-reported NPD and narcissism (PDQ-NPD: *r* = 0.45; SINS: *r* = 0.32), depression (*r* = 0.28), anxiety (*r* = 0.32), neuroticism (*r* = 0.36), and Personal Distress (*r* = 0.16), and negative associations with self-esteem (*r* = −0.25), well-being (*r* = −0.21), and agreeableness (*r* = −0.40).

**Table 5:** Bivariate correlations, similarity analyses and relative weights analysis between brief narcissism measures and external variables.

Variable		Bivariate Correlations					Relative weights				
		HSNS					HSNS					Total R^2^
		NPI-13	SB-PNI-V	OS	EGO	g-FLUX	NPI-13	SB-PNI-V	OS	EGO	g-FLUX	
Other narcissism measures	PDQ-NPD	**0.41**	*0.30*	*0.27*	*0.28*	**0.45**	0.36	0.11	0.10	0.11	0.32	*0.29*
	SINS	**0.43**	*0.21*	0.12	*0.35*	*0.32*	0.48	0.07	0.02	0.30	0.13	*0.26*
Internalizing symptoms	Depression	−0.04	**0.40**	**0.42**	0.13	*0.28*	0.05	0.36	0.40	0.03	0.16	*0.23*
	Anxiety	0.00	**0.45**	**0.45**	0.11	*0.32*	0.02	0.39	0.39	0.01	0.18	*0.27*
Well-being	RSE	0.26	**−0.53**	**−0.47**	−0.14	*−0.25*	0.23	0.41	0.24	0.02	0.09	*0.44*
	WHO	0.14	−0.33	*−0.32*	−0.07	*−0.21*	0.21	0.33	0.28	0.01	0.17	0.19
Five factor personality traits	Neuroticism	−0.13	**0.61**	**0.63**	*0.17*	*0.36*	0.09	0.37	0.40	0.02	0.12	*0.54*
	Extraversion	*0.29*	*−0.20*	*−0.28*	*−0.31*	0.04	0.30	0.10	0.15	0.37	0.07	*0.30*
	Agreeableness	**−0.45**	−0.10	−0.11	**−0.52**	**−0.40**	0.31	0.01	0.01	0.48	0.19	*0.40*
Empathy	IRI-FS	−0.06	0.12	*0.27*	−0.07	0.05	0.02	0.09	0.73	0.13	0.03	0.10
	IRI-EC	*−0.26*	<0.001	0.11	**−0.44**	−0.14	0.15	0.01	0.11	0.69	0.04	*0.27*
	IRI-PT	−0.11	0.04	0.04	*−0.26*	−0.08	0.07	0.04	0.05	0.80	0.05	0.08
	IRI-PD	−0.13	**0.43**	**0.41**	0.03	*0.16*	0.09	0.46	0.38	0.01	0.06	*0.26*
	EQ-Short	0.01	−0.07	0.02	**−0.35**	−0.06	0.03	0.04	0.06	0.84	0.03	0.16
Similarity analyses	NPI-13	**--**										
	SB-PNI-V	−0.18	--									
	HSNS OS	−0.27	***0.90***	--								
	HSNS E	**0.50** ^**^	**0.46** ^**^	0.38	--							
	g-FLUX	**0.41** ^*^	***0.79*** ^***^	***0.75*** ^***^	***0.73*** ^***^	--						

*N* = 439. Correlation coefficients above 0.16 are significant at *p* < 0.001 and in italics. Correlation coefficients above 0.40 are emboldened. Highest association with each criteria underlined. V, Vulnerability; I, Indifference/anger; G, Grandiosity/shame; A, Assertiveness/insecurity; OS, Oversensitivity to judgment; EGO, Egocentrism. rICC, Double entry q correlations.

* *p* < 0.05;

** *p* < 0.01;

*** *p* < 0.001.

To compare the overall similarities between the four brief narcissism measures, we evaluated the similarity of their correlational profiles using double entry *Q* correlations (Table 5). The NPI-13 exhibited a moderately overlapping profile with both HSNS EGO (*r_ICC_* = 0.50) *via* associations with PDQ-NPD, SINS, low agreeableness, and low Empathic Concern as well as the g-FLUX (*r_ICC_* = 0.41) *via* common associations with PDQ-NPD, SINS, and low agreeableness. By contrast, the measures of vulnerable narcissism (SB-PNI Vulnerability and HSNS OS) manifested strong overlap (*r_ICC_* = 0.90) *via* positive relationships with PDQ-NPD, depression, anxiety, neuroticism, and Personal Distress, and negative relationships with self-esteem, well-being, and extraversion. SB-PNI Vulnerability and HSNS EGO exhibited moderate similarity (*r_ICC_* = 0.46) *via* positive relationships with the PDQ-NPD, SINS, and neuroticism, and a negative relationship with extraversion. Last, although the g-FLUX demonstrated somewhat similar profiles with the NPI-13, it exhibited substantial overlap with SB-PNI Vulnerability (*r_ICC_* = 0.79) *via* positive associations with PDQ-NPD, SINS, depression, anxiety, neuroticism, Personal Distress, low self-esteem, and well-being, and OS (*r_ICC_* = 0.75) *via* positive relations to depression and anxiety, Personal Distress, lower self-esteem, and lower well-being. The g-FLUX also exhibited substantial overlap with EGO (*r_ICC_* = 0.73) *via* positive relations with PDQ-NPD, SINS, and neuroticism, and negative associations with agreeableness.

### Relative weights analyses

Lastly, we evaluated the predictive validity of the measures with the best psychometric properties (e.g., the NPI-13, SB PNI Vulnerability, HSNS OS, HSNS EGO, and the g-FLUX) by examining the relative importance of each measure as predictors of our external criteria (see Table 5). The NPI-13 was the strongest contributor to the variance in PDQ-NPD (accounting for approximately 36% of the total variance in self-reported NPD symptoms) and SINS narcissism (accounting for 48% of the total variance). SB-PNI Vulnerability was the strongest predictor of anxiety symptoms (39%), self-esteem (41%), well-being (33%), and IRI Personal Distress (46%). HSNS OS was the strongest contributors to depression (39%), anxiety (also 39%), and ISI Fantasy (73%). HSNS EGO was the strongest predictor of IRI Empathic Concern (69%), Perspective Taking (80%) and EQ Empathy (84%). The g-FLUX did not emerge as the strongest predictor of any of the outcomes but contributed approximately 32% of the variance in PDQ-NPD symptoms.

## Discussion

Decades of research on narcissism has led to the expansion of assessment tools utilized to distinguish between grandiose and vulnerable narcissism. In this study, we evaluated the psychometric properties and construct validity of four brief measures of narcissism (the NPI-13, SB-PNI, HSNS, and the g-FLUX). The current investigation enabled us to evaluate and compare their factor structure, measurement invariance across age and gender, and their construct validity in relation to several correlates of narcissism.

This is the first study, to our knowledge, to collectively evaluate these four brief narcissism instruments, including the Likert-type version of the NPI-13. Moreover, it was essential to evaluate whether the factor structure of each measure was consistent with their original versions. Considering the relationships between narcissism and variables such as age and gender, it was also important to evaluate translated assessments to verify they are measuring the same constructs in a new population. Notably, this study also provided an empirical investigation of narcissistic traits in a Finnish population where there is limited research (Ojanen et al., 2012; Annala, 2015). Finally, our examination using the g-FLUX offered additional information with respect to the construct validity of this measure in comparison to other narcissism measures and contributes to the theoretical literature on the narcissism fluctuation hypothesis (Ronningstam, 2009; Kernberg and Yeomans, 2013).

Results from our psychometric analyses suggest that the Finnish translations of the NPI-13, SB-PNI, HSNS, and g-FLUX exhibited psychometric properties that were somewhat consistent with their original versions. Consistent with our expectations, results from the CFA analyses confirmed the overall structure of the NPI-13, SB-PNI, HSNS, and the g-FLUX. Upon closer inspection of the fit indices, the NPI-13 exhibited excellent fit, high reliability, and bifactor indices to support its use as a unidimensional scale. Although the NPI-13 subscales showed good reliability consistent with previous reports (Ackerman et al., 2011; Gentile et al., 2013; Brailovskaia et al., 2019), negative item loadings on the LA and EE factors from the current study suggest some items may be problematic for subscale use alone. The SB-PNI demonstrated the best fit for the bifactor model, which is consistent with its development from the two B-PNI Grandiosity and Vulnerability composite scales (Schoenleber et al., 2015). Although the SB-PNI met criteria to support its use as a unidimensional scale, the bifactor model fit aligns with previous studies that utilize the two subfactors separately (Wright et al., 2010; Schoenleber et al., 2015). Our *post-hoc* examination of the respective SB-PNI Grandiosity and Vulnerability subfactors showed that the Vulnerability subscale demonstrated good bifactor indices, reliability, and factor loadings, whereas SB-PNI Grandiosity exhibited poor model fit and negative factor loadings. Despite previous reports that both PNI factors perform well (Wright et al., 2010), our results support the use of the SB-PNI Vulnerability subscale and suggest psychometric problems with the Grandiosity subscale. The HSNS demonstrated the closest fit with a bifactor model, which is consistent with a previous account of a two-factor solution (Fossati et al., 2009). Moreover, their relative independence from the general HSNS factor indicate that it is appropriate to utilize the OS and EGO scales separately. In comparison with HSNS OS scale, the EGO scale showed lower reliability and low item loadings. Lastly, the g-FLUX demonstrated the best fit with the correlated factor model, which is consistent with its development from items on the original FLUX scale that loaded on both the general and three subfactors (Oltmanns and Widiger, 2018). Although reliability for the general factor was high, factor loadings for the individual subfactors were slightly higher than those on the general factor. Although the bifactor indices suggest the g-FLUX can be used as a unidimensional scale, the subscales perform relatively well in comparison to the other narcissism measures. Based on the findings from our CFA analyses, our results support the psychometric soundness of the Finnish versions of the NPI-13, SB-PNI Vulnerability, HSNS OS, and the g-FLUX. Considering that we observed issues with the psychometric properties of the SB-PNI total, SB-PNI Grandiosity, the HSNS, and the HSNS EGO subscale, we recommend caution when using them in Finnish samples.

Our psychometric investigation also evaluated measurement equivalence as a function of gender and age. The NPI-13 achieved metric invariance across gender groups in this sample, meaning that the factor structure and factor loadings are similar between males and females. Moreover, partial scalar invariance could be achieved for the NPI-13 once constraints on one item from the EE subscale were lifted. Despite the limited number of males in our sample, our findings of higher latent EE scores in males aligned with previous studies suggesting that EE tends to be higher in males (Foster et al., 2003; Grijalva et al., 2015). SB-PNI-Vulnerability also achieved metric invariance for gender (as well as partial scalar invariance by relaxing constraints on two items). Similar to our results, previous studies examining gender invariance of the full-length PNI reported configural (Wright et al., 2010) and metric invariance (Jakšić et al., 2014; Morf et al., 2017). The g-FLUX also demonstrated metric invariance (with partial scalar invariance by relaxing constraints on two items). Although the configural model for HSNS did not converge, a model with OS alone demonstrated metric invariance according to gender. Overall, our findings suggest that the NPI-13, SB-PNI Vulnerability, and the g-FLUX could be examined between males and females with caution, potentially excluding the noninvariant items. Considering that the HSNS OS did not achieve scalar invariance as a function of gender (or partial scalar invariance), interpretation of means-based comparisons between males and females should proceed with caution as differences could be due to gender differences or bias in measurement.

Examination of measurement invariance as a function of age demonstrated that the NPI-13 achieved metric invariance (and partial scalar invariance) across age groups. Higher latent GE scores for younger participants in our data also align with previous reports (Hill and Roberts, 2012). Considering our data showed the pattern of decreasing NPI scores with age reported previously, we interpret the observed effects in the Finnish sample to be consistent with other investigations. SB-PNI also achieved metric invariance (and partial scalar invariance) as a function of age, which is consistent with a previous report of metric invariance using the SB-PNI (Somma et al., 2020). In the current study, we also observed decreasing SB-PNI scores as a function of age (for total scores and both composites). Our evaluation revealed that the HSNS, HSNS EGO, and the g-FLUX achieved scalar invariance as a function of age, allowing for group mean comparisons. Taken together, our findings suggest that the HSNS and g-FLUX can be examined across age groups, while the NPI-13 and SB-PNI may be examined if nonvariant items are excluded. Considering the HSNS OS only met benchmarks for metric invariance as a function of gender, interpretation of means-based comparisons between age groups should proceed with caution.

The second part of our study evaluated the construct validity of the four narcissism measures by examining their intercorrelations and associations with psychopathology symptoms, self-esteem, well-being, five factor personality traits, and empathy. This portion of our investigation focused on the best performing measures from our initial psychometric analyses (e.g., the NPI-13, SB-PNI Vulnerability, HSNS OS, HSNS EGO, and the g-FLUX). Intercorrelations between the four narcissism measures^5^ highlighted the divergence between grandiose and vulnerable traits (Fossati et al., 2009; Gentile et al., 2013; Schoenleber et al., 2015; Oltmanns and Widiger, 2018), such that the NPI-13 exhibited a modest correlation with SB-PNI Vulnerability and virtually zero correlation with HSNS OS. While two vulnerability measures were moderately interrelated (SB-PNI Vulnerability and HSNS OS), HSNS EGO exhibited a stronger relationship with the NPI-13. Consistent with previous evidence (Oltmanns and Widiger, 2018; Edershile et al., 2021), the g-FLUX was positively related to both grandiose (e.g., the NPI-13) and vulnerable narcissism (e.g., SB-PNI Vulnerability), demonstrating a slightly stronger relationship with vulnerability.

Results from the correlations and the relative weights analyses demonstrated that all four measures exhibited significant relationships with NPD symptoms and narcissistic traits. Consistent with previous reports demonstrating predictive validity of the NPI-13 (Gentile et al., 2013; Konrath et al., 2014; Krusemark et al., 2018), the NPI-13 accounted for the greatest amount of variance in self-reported PDQ-NPD symptoms. Even though all three vulnerable narcissism measures were associated with NPD symptoms, the g-FLUX and the NPI-13 was the best predictor of PDQ-NPD symptoms. Nearly all of the brief narcissism measures were related to with SINS narcissistic traits (with the exception of HSNS OS) and support previous evidence that SINS narcissism relates to grandiosity and vulnerability (Konrath et al., 2014) and highlight the utility of the NPI-13, g-FLUX, and HSNS EGO for predicting narcissistic traits. Overall, the correlational findings align with expectations that all of the narcissism would show associations with NPD as well as support previous evidence that NPD and other trait narcissism measures rely more heavily on grandiose compared to vulnerable traits (Miller et al., 2014). Moreover, the relationship between the g-FLUX and NPD symptoms provides additional support for the notion that individuals with higher levels of narcissism report more moment to moment fluctuation (Gore and Widiger, 2016; Hyatt et al., 2018), however, experimental methods using momentary assessments are best suited to address the nature of narcissistic fluctuation (Edershile et al., 2019; Edershile and Wright, 2021).

Results supported the expected pattern of higher self-esteem and well-being for the brief measure of grandiose narcissism (e.g., the NPI-13: see Rohmann et al., 2012; Gentile et al., 2013; Miller et al., 2017; Brailovskaia et al., 2019) and the contrasting pattern of diminished self-esteem, well-being, and greater psychopathology for measures of vulnerable narcissism (e.g., the SB-PNI Vulnerability and HSNS OS: see Pincus et al., 2009; Maxwell et al., 2011; Diguer et al., 2015; Brown and Brunell, 2017; Morf et al., 2017). SB-PNI Vulnerability and OS (but not EGO) exhibited associations with depression and anxiety, mirroring previous reports of increased psychopathology in vulnerable narcissism (Fossati et al., 2009; Pincus et al., 2009; Schoenleber et al., 2015). SB-PNI Vulnerability (and HSNS OS to a lesser extent) exhibited predictive power for diminished well-being and psychopathology. Lastly, the correlations between the g-FLUX, lower self-esteem, well-being, and greater psychopathology support theoretical assertions regarding narcissistic fluctuation and psychological impairment (Oltmanns and Widiger, 2018).

The five factor personality profiles were consistent with expectations for the measures of grandiose and vulnerable narcissism and further validated the personality profile of the g-FLUX. Results confirmed the extraverted, disagreeable characteristics of grandiose narcissism with the NPI-13 (Gentile et al., 2013) and supported the introverted and neurotic characteristics of vulnerable narcissism with the SB-PNI Vulnerability and HSNS OS. The personality profiles of SB-PNI Vulnerability and OS were consistent with recent research emphasizing the role of neuroticism in vulnerable narcissism (Miller et al., 2018a) and track with the observed associations between vulnerable narcissism with Axis I and II disorders, negative emotionality (Luchner and Tantleff-Dunn, 2016) and emotion regulation difficulties (Czarna et al., 2015). The personality correlates of the g-FLUX also corresponded to previous accounts of neuroticism and emotional lability (Oltmanns and Widiger, 2018), although the g-FLUX did not show the expected association with extraversion (Oltmanns and Widiger, 2018). Considering both grandiose and vulnerable narcissism share common features of antagonism in more comprehensive three-factor models of narcissism (Miller et al., 2010; Krizan and Herlache, 2018; Crowe et al., 2019), it was surprising that we did not observe correlations between all of the narcissism measures and agreeableness (only the NPI-13, HSNS EGO, and the g-FLUX were related to agreeableness). Previous evidence linking SB-PNI Vulnerability and agreeableness is mixed (Dinić and Vujić, 2019), suggesting that PNI Vulnerability may be represented by more emotional than interpersonal traits.

The correlational profiles for all four narcissism measures showed a pattern of aberrant emotional empathy. Consistent with previous reports (Hepper et al., 2014; Luchner and Tantleff-Dunn, 2016), the NPI-13 and HSNS EGO were inversely related to IRI Empathic Concern, whereas SB-PNI Vulnerability, HSNS OS, and the g-FLUX were associated with IRI Personal Distress. These findings align with reduced emotional empathy reported by individuals with grandiose and vulnerable traits (Luchner et al., 2016; Hepper et al., 2014), although it is likely that different mechanisms underlie their impairment. Grandiose individuals are less affected by others’ emotions (Czarna et al., 2015), whereas vulnerable individuals tend to experience greater emotional distress, but not concern, while observing others’ emotions (Baskin-Sommers et al., 2014; Luchner and Tantleff-Dunn, 2016). Despite previous evidence (Hepper et al., 2014), HSNS EGO was the only narcissism measure to exhibit a significant negatively relationship with cognitive aspects of empathy (Perspective Taking and EQ empathy). Although unexpected, these findings unequivocally support the assumption that self-centeredness specifically undermines the capacity to understand the thoughts of others. The pattern of diminished emotional, but not cognitive empathy supports the theory that narcissistic individuals demonstrate an unwillingness rather than an inability to empathize with others (Baskin-Sommers et al., 2014).

The study had some potential limitations. First, the sample was predominantly comprised of females and university students, which may have affected the strength of the measurement invariance findings. When interpreting our findings of gender effects in the context of previous studies, it is good to keep in mind the gender distribution. While often case in narcissism studies, especially with clinical samples, there is a lack of female participants (approximately 75% of the individuals diagnosed with NPD are males), in our sample the vast majority of participants were females. In future studies, it would be important to pay particular attention to obtaining higher number of males, as apparently the females were more likely to take the opportunity to participate to the study. Additionally, considering that all four brief narcissism measures required constraints to be lifted to achieve partial scalar invariance, it is difficult to conclude that all narcissistic traits are similar across younger and older adults in a Finnish sample (see Hill and Roberts, 2012). Although the current data demonstrated comparable construct validity to support the notion that narcissistic traits are similar between Finnish and other cultures, we did not test cultural invariance directly.

Overall, the current findings provide empirical support for the psychometric soundness and construct validity of the NPI-13, SB-PNI Vulnerability, HSNS Oversensitivity, HSNS Egocentrism, and the g-FLUX. Overall, these brief measures provide critical time savings and would be useful screeners for NPD and narcissistic fluctuation. Considering the importance of investigating all aspects of narcissistic grandiosity, vulnerability, and fluctuation between self-states, the current results demonstrate the utility of these four brief measures of narcissism.

## Data availability statement

The raw data and analysis scripts supporting the conclusions of this article will be made available by the authors, without undue reservation.

## Ethics statement

The studies involving human participants were reviewed and approved by University of Helsinki Ethical Review Board in Humanities and Social and Behavioral Sciences. The participants provided their written informed consent to participate in this study.

## Author contributions

AP and JS provided funding for the study. EK and AP oversaw project administration. PH collected and organized the data and wrote sections of the manuscript. PH and EK performed the statistical analysis and created tables for the manuscript. EK wrote the first draft of the manuscript. PH, JS, AP, and EK contributed to the conceptualization and methodological design of the study. All authors contributed to the article and approved the submitted version.

## Funding

The study was supported by the Academy of Finland (grants #319113 and #320248 to AP, grants #325981 and #328954 to JS).

## Conflict of interest

The authors declare that the research was conducted in the absence of any commercial or financial relationships that could be construed as a potential conflict of interest.

## Publisher’s note

All claims expressed in this article are solely those of the authors and do not necessarily represent those of their affiliated organizations, or those of the publisher, the editors and the reviewers. Any product that may be evaluated in this article, or claim that may be made by its manufacturer, is not guaranteed or endorsed by the publisher.

## Footnotes

^1^We also examined the diagnostic potential of the four narcissism scales in predicting NPD symptoms and self-reported narcissistic traits using receiver operating characteristic (ROC) analyses (see Supplementary material).

^2^In a post-hoc analysis, fit indices of the single factor SB-PNI-Grandiosity were below satisfactory (CFI = 0.751, TLI = 0.585, RMSEA = 0.312, SRMR = 0.151), whereas the parameters of the single factor SB-PNI-Vulnerability were excellent (CFI = 0.958, TLI = 0.930, SRMR = 0.057) apart from a less satifactory RMSEA score (0.135).

^3^We continued this portion of our investigation by focusing on the best performing measures from our initial psychometric analyses (e.g., the NPI-13, SB-PNI Vulnerability, HSNS OS, HSNS EGO, and the g-FLUX).

^4^The average intercorrelation with the four original scales (e.g., the NPI-13, SB-PNI Total, HSNS Total, and the g-FLUX) was 0.40.

^5^Due to inadequate psychometric properties for the SB-PNI total and SB-PNI Grandiosity, and the HSNS, we did not discuss their intercorrelations or associations with additional variables. Nonetheless, our data demonstrated the expected positive correlations between the NPI-13 and SB-PNI total (r = 0.28) and SB-PNI Grandiosity (r = 0.35) as well as positive correlations between the HSNS and SB-PNI total (r = 0.51) and SB-PNI Vulnerability (r = 0.55).

## References

[ref1] Ackerman R. A. Witt E. A. Donnellan M. B. Trzesniewski K. H. Robins R. W. Kashy D. A. (2011). What does the narcissistic personality inventory really measure? Assessment 18, 67–87. doi: 10.1177/1073191110382845 20876550

[ref2] American Psychiatric Association (1980). Diagnostic and statistical manual of mental disorders. *3rd Edn.* Washington, DC: American Psychiatric Association.

[ref3] Annala M. (2015). Aggressiivisempi ihminen: Syynulkoistamistaipumus välittämässä häpeäherkkyyden ja narsismin yhteyksiä aggressiivisuuteen. master’s thesis. University of Helsinki.

[ref4] Baskin-Sommers A. Krusemark E. Ronningstam E. (2014). Empathy in narcissistic personality disorder: from clinical and empirical perspectives. Personal. Disord. Theory Res. Treat. 5, 323–333. doi: 10.1037/per0000061, PMID: 24512457 PMC4415495

[ref5] Beauducel A. Herzberg P. Y. (2006). On the performance of maximum likelihood versus means and variance adjusted weighted least squares estimation in CFA. Struct. Equ. Modeling 13, 186–203. doi: 10.1207/s15328007sem1302_2

[ref6] Brailovskaia J. Bierhoff H. W. Margraf J. (2019). How to identify narcissism with 13 items? Validation of the German narcissistic personality inventory–13 (G-NPI-1 3). Assessment 26, 630–644. doi: 10.1177/1073191117740625, PMID: 29117708

[ref7] Brown A. A. Brunell A. B. (2017). The “modest mask”? An investigation of vulnerable narcissists’ implicit self-esteem. Personal. Individ. Differ. 119, 160–167. doi: 10.1016/j.paid.2017.07.020

[ref8] Bushman B. J. Baumeister R. F. (1998). Threatened egotism, narcissism, self-esteem, and direct and displaced aggression: does self-love or self-hate lead to violence? J. Pers. Soc. Psychol. 75, 219–229. doi: 10.1037/0022-3514.75.1.219, PMID: 9686460

[ref9] Campbell W. K. Foster C. A. Finkel E. J. (2002). Does self-love lead to love for others? A story of narcissistic game playing. J. Pers. Soc. Psychol. 83, 340–354. doi: 10.1037/0022-3514.83.2.340, PMID: 12150232

[ref10] Cozma I. Javadian G. Gupta V. K. Canever M. (2014). Narcissistic personality inventory: an assessment of measurement equivalence across countries and gender. Int. J. Manage. Bus. 5, 105–121.

[ref11] Crowe M. Lynam D. R. Miller J. D. (2019). Exploring the structure of narcissism: towards an integrated solution. J. Pers. 87, 1151–1169. doi: 10.1111/jopy.12464, PMID: 30742713

[ref12] Czarna A. Z. Wróbel M. Dufner M. Zeigler-Hill V. (2015). Narcissism and emotional contagion: do narcissists “catch” the emotions of others? Soc. Psychol. Personal. Sci. 6, 318–324. doi: 10.1177/1948550614559652

[ref13] Davis M. H. (1983). Measuring individual differences in empathy: evidence for a multidimensional approach. J. Pers. Soc. Psychol. 44, 113–126. doi: 10.1037/0022-3514.44.1.113

[ref14] Diguer L. Turmel V. da Silva R. L. Mathieu V. (2015). Convergent and clinical validity of the pathological narcissism inventory. J. Am. Psychoanal. Assoc. 63, NP1–NP3. doi: 10.1177/0003065115604652, PMID: 26487118

[ref15] Dinić B. M. Vujić A. (2019). The pathological narcissism inventory: measurement invariance across Serbian and USA samples and further validation. Eur. J. Psychol. Assess. 670–680. doi: 10.1027/1015-5759/a000537

[ref16] Edershile E. A. Oltmanns J. R. Widiger T. A. Wright A. G. (2021). Predicting fluctuation in narcissistic states: An examination of the g-FLUX scale Psychol. Assess. 33:60. 10.1037/pas0000967 PMC8452271 33090828

[ref17] Edershile E. A. Woods W. C. Sharpe B. M. Crowe M. L. Miller J. D. Wright A. G. C. (2019). A day in the life of narcissus: measuring narcissistic grandiosity and vulnerability in daily life. Psychol. Assess. 31, 913–924. doi: 10.1037/pas0000717, PMID: 30883153

[ref18] Edershile E. A. Wright A. G. C. (2021). Fluctuations in grandiose and vulnerable narcissistic states: a momentary perspective. J. Pers. Soc. Psychol. 120, 1386–1414. doi: 10.1037/pspp0000370, PMID: 33090821 PMC8060359

[ref19] Eid M. Geiser C. Koch T. Heene M. (2017). Anomalous results in G-factor models: explanations and alternatives. Psychol. Methods 22, 541–562. doi: 10.1037/met0000083, PMID: 27732052

[ref20] Fossati A. Borroni S. Grazioli F. Dornetti L. Marcassoli I. Maffei C.. (2009). Tracking the hypersensitive dimension in narcissism: reliability and validity of the hypersensitive narcissism scale. Personal. Ment. Health 3, 235–247. doi: 10.1002/pmh.92

[ref21] Foster J. D. Campbell W. K. Twenge J. M. (2003). Individual differences in narcissism: inflated self-views across the lifespan and around the world. J. Res. Pers. 37, 469–486. doi: 10.1016/S0092-6566(03)00026-6

[ref22] Gentile B. Miller J. D. Hoffman B. J. Reidy D. E. Zeichner A. Campbell W. K. (2013). A test of two brief measures of grandiose narcissism: the narcissistic personality inventory–13 and the narcissistic personality Inventory-16. Psychol. Assess. 25, 1120–1136. doi: 10.1037/a0033192, PMID: 23815119

[ref23] Giacomin M. Jordan C. H. (2016). Self-focused and feeling fine: assessing state narcissism and its relation to well-being. J. Res. Pers. 63, 12–21. doi: 10.1016/j.jrp.2016.04.009

[ref24] Gore W. L. Widiger T. A. (2016). Fluctuation between grandiose and vulnerable narcissism. Personal. Disord. Theory Res. Treat. 7, 363–371. doi: 10.1037/per0000181, PMID: 26986960

[ref25] Grijalva E. Newman D. A. Tay L. Donnellan M. B. Harms P. D. Robins R. W.. (2015). Gender differences in narcissism: a meta-analytic review. Psychol. Bull. 141, 261–310. doi: 10.1037/a0038231, PMID: 25546498

[ref26] Hendin H. M. Cheek J. M. (1997). Assessing hypersensitive narcissism: a reexamination of Murray's Narcism scale. J. Res. Pers. 31, 588–599. doi: 10.1006/jrpe.1997.2204

[ref27] Hepper E. G. Hart C. M. Meek R. Cisek S. Sedikides C. (2014). Narcissism and empathy in young offenders and non-offenders. Eur. J. Pers. 28, 201–210. doi: 10.1002/per.1939

[ref03] Hill P. L. Roberts B. W. (2012). Narcissism, well-being, and observer-rated personality across the lifespan. Soc. Psychol. Personal. Sci. 3, 216–223.

[ref28] Holtzman N. S. Vazire S. Mehl M. R. (2010). Sounds like a narcissist: behavioral manifestations of narcissism in everyday life. J. Res. Pers. 44, 478–484. doi: 10.1016/j.jrp.2010.06.001, PMID: 20711512 PMC2918908

[ref29] Hu L. T. Bentler P. M. (1999). Cutoff criteria for fit indexes in covariance structure analysis: conventional criteria versus new alternatives. Struct. Equ. Model. Multidiscip. J. 6, 1–55. doi: 10.1080/10705519909540118

[ref30] Hyatt C. S. Sleep C. E. Lynam D. R. Widiger T. A. Campbell W. K. Miller J. D. (2018). Ratings of affective and interpersonal tendencies differ for grandiose and vulnerable narcissism: a replication and extension of Gore & Widiger (2016). J. Pers. 86, 422–434. doi: 10.1111/jopy.12325, PMID: 28509415

[ref31] Hyler S. E. (1994). Personality diagnostic questionnaire–4. New York, NY: New York State Psychiatric Institute.

[ref32] Jakšić N. Milas G. Ivezić E. Wertag A. Jokić-Begić N. Pincus A. L. (2014). The pathological narcissism inventory (PNI) in transitional post-war Croatia: psychometric and cultural considerations. J. Psychopathol. Behav. Assess. 36, 640–652. doi: 10.1007/s10862-014-9425-2

[ref33] Johnson J. W. (2000). A heuristic method for estimating the relative weight of predictor variables in multiple regression. Multivar. Behav. Res. 35, 1–19. doi: 10.1207/S15327906MBR3501_1, PMID: 26777229

[ref34] Kalliopuska M. (1992). Self-esteem and narcissism among the most and least empathetic Finnish baseball players. Percept. Mot. Skills 75, 945–946. doi: 10.2466/pms.1992.75.3.945, PMID: 1454500

[ref35] Kernberg P. F. (1998). “Developmental aspects of normal and pathological narcissism,” in Disorders of narcissism: Diagnostic, clinical, and empirical implications. ed. Ronningstam E. F. (Washington, D.C: American Psychiatric Press), 103–120.

[ref36] Kernberg O. F. Yeomans F. E. (2013). Borderline personality disorder, bipolar disorder,depression, attention deficit/hyperactivity disorder, and narcissistic personality disorder: practical differential diagnosis. Bull. Menninger Clin. 77, 1–22. doi: 10.1521/bumc.2013.77.1.1, PMID: 23428169

[ref37] Konrath S. Meier B. P. Bushman B. J. (2014). Development and validation of the single item narcissism scale (SINS). PLoS One 9:e103469. doi: 10.1371/journal.pone.0103469, PMID: 25093508 PMC4122388

[ref38] Konstabel K. Lönnqvist J. E. Walkowitz G. Konstabel K. Verkasalo M. (2012). The ‘short five’(S5): measuring personality traits using comprehensive single items. Eur. J. Pers. 26, 13–29. doi: 10.1002/per.813

[ref39] Krizan Z. Herlache A. D. (2018). The narcissism spectrum model: a synthetic view of narcissistic personality. Pers. Soc. Psychol. Rev. 22, 3–31. doi: 10.1177/1088868316685018, PMID: 28132598

[ref40] Kroenke K. Spitzer R. L. Williams J. B. (2001). The PHQ-9: validity of a brief depression severity measure. J. Gen. Intern. Med. 16, 606–613. doi: 10.1046/j.1525-1497.2001.016009606.x, PMID: 11556941 PMC1495268

[ref41] Krusemark E. A. Campbell W. K. Crowe M. L. Miller J. D. (2018). Comparing self-report measures of grandiose narcissism, vulnerable narcissism, and narcissistic personality disorder in a male offender sample. Psychol. Assess. 30, 984–990. doi: 10.1037/pas0000579, PMID: 29792507

[ref42] Li C. H. (2016). The performance of ML, DWLS, and ULS estimation with robust corrections in structural equation models with ordinal variables. Psychol. Methods 21, 369–387. doi: 10.1037/met0000093, PMID: 27571021

[ref43] Luchner A. F. Tantleff-Dunn S. (2016). Dysfunctional empathy in vulnerable narcissism. N. Am. J. Psychol. 18, 597–610.

[ref44] Mardia K. V. (1970). Measures of multivariate skewness and kurtosis with applications. Biometrika 57, 519–530. doi: 10.1093/biomet/57.3.519

[ref45] Maxwell K. Donnellan M. B. Hopwood C. J. Ackerman R. A. (2011). The two faces of narcissus? An empirical comparison of the narcissistic personality inventory and the pathological narcissism inventory. Personal. Individ. Differ. 50, 577–582. doi: 10.1016/j.paid.2010.11.031

[ref01] McCrae R. R. (2008). A note on some measures of profile agreement. J. Pers. Assess. 90, 105–109. 18444102 10.1080/00223890701845104 PMC5929980

[ref46] Miller J. D. Campbell W. K. Pilkonis P. A. (2007). Narcissistic personality disorder: relations with distress and functional impairment. Compr. Psychiatry 48, 170–177. doi: 10.1016/j.comppsych.2006.10.003, PMID: 17292708 PMC1857317

[ref47] Miller J. D. Dir A. Gentile B. Wilson L. Pryor L. R. Campbell W. K. (2010). Searching for a vulnerable dark triad: comparing factor 2 psychopathy, vulnerable narcissism, and borderline personality disorder. J. Pers. 78, 1529–1564. doi: 10.1111/j.1467-6494.2010.00660.x, PMID: 20663024

[ref48] Miller J. D. Gentile B. Carter N. T. Crowe M. Hoffman B. J. Campbell W. K. (2018b). A comparison of the nomological networks associated with forced-choice and likert formats of the narcissistic personality inventory. J. Pers. Assess. 100, 259–267. doi: 10.1080/00223891.2017.1310731, PMID: 28436690

[ref02] Miller J. D. Lynam D. R. Hyatt C. S. Campbell W. K. (2017). Controversies in narcissism. Annu. Rev. Clin. Psychol. 13, 291–315. 28301765 10.1146/annurev-clinpsy-032816-045244

[ref49] Miller J. D. Lynam D. R. Vize C. Crowe M. Sleep C. Maples-Keller J. L.. (2018a). Vulnerable narcissism is (mostly) a disorder of neuroticism. J. Pers. 86, 186–199. doi: 10.1111/jopy.12303, PMID: 28170100

[ref50] Miller J. D. McCain J. Lynam D. R. Few L. R. Gentile B. MacKillop J.. (2014). A comparison of the criterion validity of popular measures of narcissism and narcissistic personality disorder via the use of expert ratings. Psychol. Assess. 26, 958–969. doi: 10.1037/a0036613, PMID: 24773036

[ref51] Morf C. C. Rhodewalt F. (2001). Unraveling the paradoxes of narcissism: a dynamic self- regulatory processing model. Psychol. Inq. 12, 177–196. doi: 10.1207/S15327965PLI1204_1

[ref52] Morf C. C. Schürch E. Küfner A. Siegrist P. Vater A. Back M.. (2017). Expanding the nomological net of the pathological narcissism inventory: German validation and extension in a clinical inpatient sample. Assessment 24, 419–443. doi: 10.1177/1073191115627010, PMID: 26874362

[ref53] Ojanen T. Findley D. Fuller S. (2012). Physical and relational aggression in early adolescence: associations with narcissism, temperament, and social goals. Aggress. Behav. 38, 99–107. doi: 10.1002/ab.21413, PMID: 22331610

[ref54] Oltmanns J. R. Widiger T. A. (2018). Assessment of fluctuation between grandiose and vulnerable narcissism: development and initial validation of the FLUX scales. Psychol. Assess. 30, 1612–1624. doi: 10.1037/pas0000616, PMID: 29927302 PMC6265070

[ref55] Paunonen S. V. Lönnqvist J. E. Verkasalo M. Leikas S. Nissinen V. (2006). Narcissism and emergent leadership in military cadets. Leadersh. Q. 17, 475–486. doi: 10.1016/j.leaqua.2006.06.003

[ref56] Pechorro P. Maroco J. Ray J. V. Gonçalves R. A. Nunes C. (2018). A brief measure of narcissism among female juvenile delinquents and community youths: the narcissistic personality inventory–13. Int. J. Offender Ther. Comp. Criminol. 62, 2292–2311. doi: 10.1177/0306624X17700855, PMID: 28355931

[ref57] Pincus A. L. Ansell E. B. Pimentel C. A. Cain N. M. Wright A. G. Levy K. N. (2009). Initial construction and validation of the pathological narcissism inventory. Psychol. Assess. 21, 365–379. doi: 10.1037/a0016530, PMID: 19719348

[ref58] Pincus A. L. Lukowitsky M. R. (2010). Pathological narcissism and narcissistic personality disorder. Annu. Rev. Clin. Psychol. 6, 421–446. doi: 10.1146/annurev.clinpsy.121208.131215 20001728

[ref59] Putnick D. L. Bornstein M. H. (2016). Measurement invariance conventions and reporting: the state of the art and future directions for psychological research. Dev. Rev. 41, 71–90. doi: 10.1016/j.dr.2016.06.004, PMID: 27942093 PMC5145197

[ref60] Raskin R. N. Hall C. S. (1979). A narcissistic personality inventory. Psychol. Rep. 45:590. doi: 10.2466/pr0.1979.45.2.590 538183

[ref61] Reise S. P. Scheines R. Widaman K. F. Haviland M. G. (2013). Multidimensionality and structural coefficient bias in structural equation modeling: a bifactor perspective. Educ. Psychol. Meas. 73, 5–26. doi: 10.1177/0013164412449831

[ref62] Rodriguez A. Reise S. P. Haviland M. G. (2016). Applying bifactor statistical indices in the evaluation of psychological measures. J. Pers. Assess. 98, 223–237. doi: 10.1080/00223891.2015.1089249, PMID: 26514921

[ref63] Rohmann E. Neumann E. Herner M. J. Bierhoff H. W. (2012). Grandiose and vulnerable narcissism: self-construal, attachment, and love in romantic relationships. Eur. Psychol. 17, 279–290. doi: 10.1027/1016-9040/a000100

[ref64] Ronningstam E. (2009). Narcissistic personality disorder: facing DSM-V. Psychiatr. Ann. 39, 111–121. doi: 10.3928/00485713-20090301-09

[ref65] Rosenberg M. (1965). Society and the adolescent self-image. Princeton, NJ: Princeton University Press.

[ref66] Rosseel Y. (2012). Lavaan: an R package for structural equation modeling and more. J. Stat. Softw. 48, 1–36. doi: 10.18637/jss.v048.i02

[ref67] Schoenleber M. Roche M. J. Wetzel E. Pincus A. L. Roberts B. W. (2015). Development of a brief version of the pathological narcissism inventory. Psychol. Assess. 27, 1520–1526. doi: 10.1037/pas0000158, PMID: 26011478 PMC4659764

[ref68] Sedikides C. Rudich E. A. Gregg A. P. Kumashiro M. Rusbult C. (2004). Are normal narcissists psychologically healthy? Self-esteem matters. J. Pers. Soc. Psychol. 87, 400–416. doi: 10.1037/0022-3514.87.3.400, PMID: 15382988

[ref69] Somma A. Pincus A. L. Fontana A. Cianfanelli B. Fossati A. (2020). Measurement invariance of three versions of the pathological narcissism inventory across gender-matched Italian adolescent high school and young adult university students. J. Psychopathol. Behav. Assess. 42, 38–51. doi: 10.1007/s10862-019-09758-7

[ref70] Spitzer R. L. Kroenke K. Williams J. B. Löwe B. (2006). A brief measure for assessing generalized anxiety disorder: the GAD-7. Arch. Intern. Med. 166, 1092–1097. doi: 10.1001/archinte.166.10.1092, PMID: 16717171

[ref71] Steinmetz H. (2013). Analyzing observed composite differences across groups: is partial measurement invariance enough? Methodology 9, 1–12. doi: 10.1027/1614-2241/a000049

[ref72] Tonidandel S. LeBreton J. M. (2011). Relative importance analysis: a useful supplement to regression analysis. J. Bus. Psychol. 26, 1–9. doi: 10.1007/s10869-010-9204-3

[ref73] Topp C. W. Østergaard S. D. Søndergaard S. Bech P. (2015). The WHO-5 well-being index: a systematic review of the literature. Psychother. Psychosom. 84, 167–176. doi: 10.1159/000376585, PMID: 25831962

[ref74] Wakabayashi A. Baron-Cohen S. Wheelwright S. Goldenfeld N. Delaney J. Fine D.. (2006). Development of short forms of the empathy quotient (EQ-short) and the systemizing quotient (SQ-short). Personal. Individ. Differ. 41, 929–940. doi: 10.1016/j.paid.2006.03.017

[ref75] Wink P. (1991). Two faces of narcissism. J. Pers. Soc. Psychol. 61, 590–597. doi: 10.1037/0022-3514.61.4.590 1960651

[ref76] Wright A. G. Lukowitsky M. R. Pincus A. L. Conroy D. E. (2010). The higher order factor structure and gender invariance of the pathological narcissism inventory. Assessment 17, 467–483. doi: 10.1177/1073191110373227, PMID: 20634422

