# Supplementary material for: Grandiosity, vulnerability, and narcissistic fluctuation: Examining reliability, measurement invariance, and construct validity of four brief narcissism measures
Source: Front Psychol. 2022 Oct 10;13:993663. doi: 10.3389/fpsyg.2022.993663 (PMC9589046; doi:10.3389/fpsyg.2022.993663)

| **Supplementary Table 1. Descriptive Information.** | | | | | | | | | | |
| --- | --- | --- | --- | --- | --- | --- | --- | --- | --- | --- |
| **Variable** |  | n | **Items** | **Scale** | | **Range** | **Mean (SD)** | **α** | **Skewness** | **Kurtosis** |
| Age |  | 439 |  |  | | 19 - 67 | 28.81 (8.85) |  | 1.68 | 2.79 |
| Education | Level (4 = BA) | 439 |  | 1-6 | | 2 - 6 | 3.78 (0.81) |  |  |  |
| **Narcissism measures** | | | | |  | | | | | |
|  | NPI-13 total | 439 | 13 | 1 - 5 | | 13 - 57 | 31.30 (8.47) | 0.84 | 0.31 | -0.21 |
|  | Grandiose Exhibitionism (GE) | 439 | 5 |  |  | 5 - 23 | 11.64 (3.98) | 0.75 | 0.32 | -0.60 |
|  | Leadership/Authority (LA) | 439 | 4 |  |  | 4 - 19 | 8.85 (3.34) | 0.77 | 0.54 | -0.23 |
|  | Entitlement/Exploitativeness (EE) | 439 | 4 |  |  | 4 - 19 | 10.80 (3.14) | 0.63 | 0.20 | -0.30 |
|  | SB-PNI Total | 439 | 12 | 0 - 5 | | 0 - 57 | 31.02 (10.22) | 0.84 | -0.11 | -0.23 |
|  | SB-PNI-Grandiosity (SB-PNI-G) | 439 | 6 |  |  | 0 - 30 | 17.65 (5.41) | 0.72 | -0.32 | -0.09 |
|  | SB-PNI-Vulnerability (SB-PNI-V) | 439 | 6 |  |  | 0 - 29 | 13.37 (6.29) | 0.81 | 0.01 | -0.65 |
|  | Hypersensitive Narcissism (HSNS) | 439 | 10 | 1 - 5 | | 15 - 50 | 31.37 (5.62) | 0.69 | 0.07 | 0.22 |
|  | g-FLUX total | 439 | 9 | 1 - 5 | | 9 - 45 | 25.30 (7.34) | 0.84 | -0.02 | -0.53 |
|  | Indifference/Anger (I) | 439 | 3 |  |  | 3 - 15 | 8.03 (2.83) | 0.69 | 0.06 | -0.68 |
|  | Grandiosity/Shame (G) | 439 | 3 |  |  | 3 - 15 | 8.85 (3.37) | 0.80 | -0.08 | -0.99 |
|  | Assertiveness/Insecurity (A) | 439 | 3 |  |  | 3 - 15 | 8.42 (2.69) | 0.65 | 0.08 | -0.50 |
| **Other Narcissism Measures** | | | | | | | | | | |
|  | PDQ-NPD symptoms | 439 | 9 | 0 - 1 | | 0 - 7 | 2.04 (1.50) | 0.42 | 0.68 | 0.22 |
|  | Single item narcissism (SINS) | 439 | 1 | 1 - 7 | | 1 - 6 | 2.03 (1.18) | -- | 1.16 | 0.53 |
| **Psychopathology Symptoms** | | | | | | | | | | |
|  | Depression (PHQ-9) | 439 | 9 | 0 - 3 | | 0 - 27 | 7.41 (6.09) | 0.89 | 0.95 | 0.21 |
|  | Anxiety (GAD-7) | 439 | 7 | 0 - 3 | | 0 -27 | 5.98 (4.59) | 0.87 | 1.03 | 0.65 |
| **Well-Being Indices** | | | | | | | | | | |
|  | Rosenberg Self-Esteem (RSE) | 439 | 10 | 1 - 5 | | 12 - 50 | 33.68 (8.14) | 0.90 | -0.37 | -0.36 |
|  | Well-being (WHO-5) | 439 | 5 | 0 - 5 | | 0 - 24 | 13.17 (5.10) | 0.88 | -0.06 | -0.62 |
| **Five Factor personality** | | | | | | | | | | |
|  | Short Five Neuroticism (N) | 438 | 12 | -3 - 3 | | -32 - 30 | -1.26 (13.66) | 0.82 | 0.01 | -0.53 |
|  | Extraversion (E) | 438 | 12 |  |  | -34 - 32 | 1.82 (12.55) | 0.79 | -0.03 | -0.60 |
|  | Agreeableness (A) | 438 | 12 |  |  | -20 - 36 | 15.10 (9.67) | 0.71 | -0.70 | 0.45 |
| **Empathy** | | | | | | | | | | |
|  | Interpersonal Reactivity Index (IRI)  Fantasy (FS) | 439 | 7 | 0 - 4 | | 5 - 28 | 19.84 (5.05) | 0.81 | -0.64 | -0.05 |
|  | Empathic concern (EC) | 439 | 7 |  |  | 2 - 28 | 19.56 (4.79) | 0.81 | -0.79 | 0.63 |
|  | Perspective taking (PT) | 439 | 7 |  |  | 4 - 28 | 18.50 (4.28) | 0.74 | -0.45 | 0.44 |
|  | Personal distress (PD) | 439 | 7 |  |  | 0 - 26 | 12.44 (5.18) | 0.79 | 0.06 | -0.37 |
|  | Empathy Quotient (EQ-Short) | 439 | 22 | 0 - 2 | | 0 - 44 | 24.03 (8.73) | 0.90 | -0.27 | -0.57 |

**Supplementary Table 2. Intercorrelations between brief narcissism scales, subscales, and age.**

|  | Variable | **1** | 2 | 3 | 4 | **5** | 6 | 7 | **8** | 9 | 10 | **11** | 12 | 13 | 14 |
| --- | --- | --- | --- | --- | --- | --- | --- | --- | --- | --- | --- | --- | --- | --- | --- |
| **NPI-13 total** | **1** | (.29) |  |  |  |  |  |  |  |  |  |  |  |  |  |
| Leadership/Authority (LA) | 2 | ***.83*** | (.45) |  |  |  |  |  |  |  |  |  |  |  |  |
| Grandiose/Exhibitionism (GE) | 3 | ***.79*** | *.42* | (.37) |  |  |  |  |  |  |  |  |  |  |  |
| Entitlement/Exploitativeness (EE) | 4 | ***.82*** | ***.65*** | *.40* | (.30) |  |  |  |  |  |  |  |  |  |  |
| **SB-PNI Total** | **5** | **.28** | .18 | .21 | .29 | (.30) |  |  |  |  |  |  |  |  |  |
| SB-PNI Grandiosity | 6 | *.35* | *.32* | .23 | *.33* | ***.85*** | (.30) |  |  |  |  |  |  |  |  |
| PNI Vulnerability | 7 | .14 | .01 | .15 | .19 | ***.89*** | ***.52*** | (.42) |  |  |  |  |  |  |  |
| **HSNS Total** | **8** | **.16** | .02 | .12 | .25 | ***.51*** | *.31* | ***.55*** | (.17) |  |  |  |  |  |  |
| Oversensitivity to Judgment (OS) | 9 | .03 | -.09 | .06 | .10 | ***.55*** | *.34* | ***.60*** | ***.83*** | (.25) |  |  |  |  |  |
| Egocentrism (EGO) | 10 | .22 | .14 | .12 | .29 | .16 | .10 | .17 | ***.72*** | *.25* | (.26) |  |  |  |  |
| **g-FLUX Total** | **11** | ***.43*** | *.32* | .25 | *.49* | ***.56*** | *.47* | ***.50*** | *.49* | *.39* | .35 | (.36) |  |  |  |
| Indifference/Anger (I) | 12 | *.32* | .27 | .15 | *.39* | .27 | .22 | .25 | *.34* | .23 | .28 | ***.76*** | (.43) |  |  |
| Grandiosity/Shame (G) | 13 | *.32* | .21 | .20 | *.37* | ***.62*** | ***.54*** | ***.54*** | *.46* | *.39* | .29 | ***.86*** | *.42* | (.57) |  |
| Assertiveness/Insecurity (A) | 14 | *.43* | *.32* | .28 | *.46* | *.46* | *.37* | *.42* | *.41* | *.33* | .28 | ***.85*** | *.50* | ***.64*** | (.39) |
| **Age** | **15** | -.14 | -.08 | -.16 | -.09 | -.28 | -.29 | -.21 | -.11 | -.15 | .00 | -.08 | -.05 | -.18 | -.09 |

Note: N=439. Average inter-item correlation on diagonal in parentheses. Coefficients above 0.10 are significant at p<0.05. Coefficients above 0.16 are significant at p<0.001. Coefficients 0.30 or above diagonal, coefficients ≥0.50 in bold.

**Supplementary Table 3. Independent invariance test fit indices between configural, metric and scalar models of HSNS OS and EGO subfactors in terms of gender and age.**

| **Variable** |  | **Model** | **df** | **χ^2^** | **Comp. to** | **p(χ2)** | **CFI** | **TLI** | **RMSEA** | **SRMR** |
| --- | --- | --- | --- | --- | --- | --- | --- | --- | --- | --- |
| Gender | OS | Configural | 10 | 41.79 |  |  | .945 | .89 | .123 | .064 |
|  |  | Metric | 14 | 39.685 | Configural | .410 | .956 | .937 | .093 | .068 |
|  |  | Scalar | 28 | 96.36 | Metric | **<.001***** | .882 | .916 | .107 | .074 |
|  | EGO | Configural | nc |  |  |  |  |  |  |  |
|  |  |  |  |  |  |  |  |  |  |  |
| Age | OS | Configural | 10 | 33.76 |  |  | .968 | .935 | .104 | .055 |
|  |  | Metric | 14 | 51.46 | Configural | **<.001***** | .949 | .927 | .111 | .074 |
|  |  | Scalar | 28 | 78.41 | Metric | .061 | .931 | .951 | .091 | .069 |
|  | EGO | Configural | 4 | 10.60 |  |  | .976 | .929 | .087 | .036 |
|  |  | Metric | 7 | 10.98 | Configural | .811 | .986 | .976 | .051 | .038 |
|  |  | Scalar | 18 | 25.34 | Metric | .244 | .974 | .983 | .043 | .039 |

**Supplemental Figure 1**. ROC curves of narcissism scales predicting NPD (A) and agreeing to SINS statement (B) with area under curve (AUC) statistics and 95% confidence intervals.

1. PDQ-SYMPTOM SCORE >4, (DSM-V DIAGNOSIS CRITERIA)


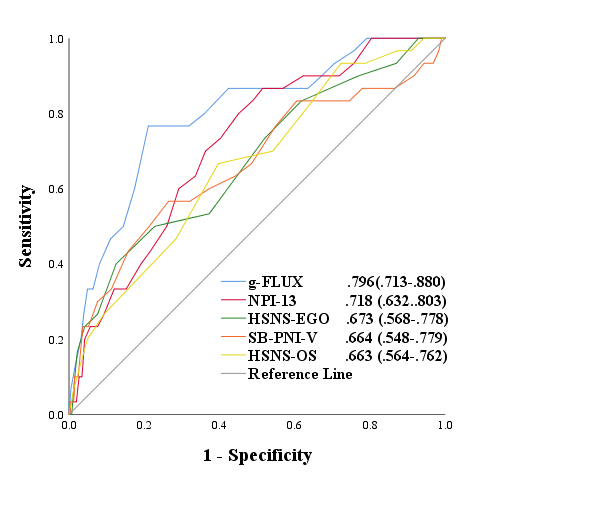


1. SINS SCORE > 4 (AGREE ABOVE MEAN TO THE STATEMENT)


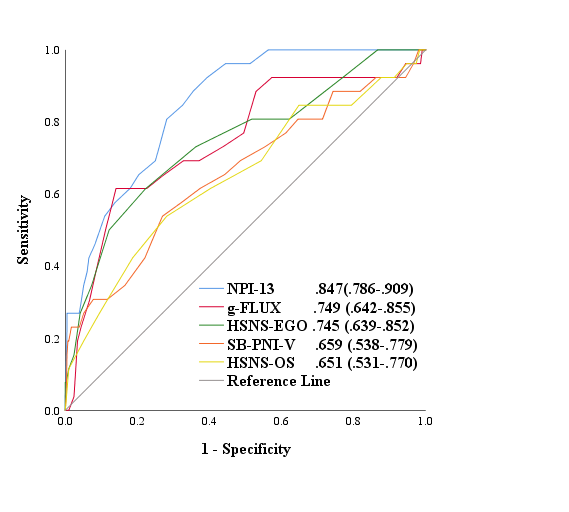

Supplement: Supplementary file 1 [file Table_1.DOCX]
